# Exploring the Mechanism of *Scutellaria baicalensis* Georgi Efficacy against Oral Squamous Cell Carcinoma Based on Network Pharmacology and Molecular Docking Analysis

**DOI:** 10.1155/2021/5597586

**Published:** 2021-07-13

**Authors:** Fanfan Hou, Yang Liu, YaHsin Cheng, Ni Zhang, Wenpeng Yan, Fang Zhang

**Affiliations:** ^1^Stomatology Hospital, Shanxi Medical University, Taiyuan 030001, China; ^2^Department of Physiology, School of Medicine, China Medical University, Taichung, Taiwan

## Abstract

**Background:**

*Scutellaria baicalensis* Georgi (SBG) has been widely shown to induce apoptosis and inhibit invasion and migration of various cancer cells. Increased evidence shows that SBG may be useful to treat oral squamous cell carcinoma (OSCC). However, the biological activity and possible mechanisms of SBG in the treatment of OSCC have not been fully elucidated. This study aimed to clarify the bioactive component and multitarget mechanisms of SBG against OSCC using network pharmacology and molecular docking.

**Methods:**

Traditional Chinese Medicine Systems Pharmacology (TCMSP) database was used to predict the active components in SBG, and putative molecular targets of SBG were identified using the Swiss Target Prediction database. OSCC-related targets were screened by GeneCards, Online Mendelian Inheritance in Man (OMIM), and Therapeutic Target Database (TTD). Then, we established protein-protein interaction (PPI), compound-target-disease (C-T-D), and compound-target-pathway (C-T-P) networks by Cytoscape to identify the main components, core targets, and pharmacological pathways of SBG against OSCC via applying data mining techniques and topological parameters. Metascape database was utilized for Gene Ontology (GO) and pathway enrichment analysis. The potential interaction of the main components with core targets was revealed by molecular docking simulation, and for the correlation between core targets and OSCC prognosis analysis, the Kaplan–Meier Plotter online database was used.

**Results:**

There were 25 active compounds in SBG and 86 genes targeted by OSCC. A total of 141 signaling pathways were identified, and it was found that the PI3K-Akt signaling pathway may occupy core status in the anti-OSCC system. GO analysis revealed that the primary biological processes were related to apoptosis, proliferation, and migration. Molecular docking results confirmed that core targets of OSCC had a high affinity with the main compounds of SBG.

**Conclusion:**

Our study demonstrated multicomponent, multitarget, and multipathway characteristics of SBG in the treatment of OSCC and provided a foundation for further drug development research.

## 1. Introduction

Oral squamous cell carcinoma (OSCC) is one of the most ubiquitous head and neck cancers and is prevalent worldwide, with about 350,000 newly diagnosed patients in 2018 [1]. Tobacco use, human papillomavirus (HPV), and alcohol consumption are considered the independent risk factors for OSCC [2]. Currently, OSCC requires a combination of treatments, including surgery, chemotherapy, radiotherapy, and immunotherapy [3]. Despite numerous attempts with different chemotherapy regimens and molecular target-based therapies, the overall 5-year survival rate of OSCC remains at 50%–60%. However, chemotherapy for patients who cannot undergo surgical resection may prolong survival by about 15% while leading to the undesirable side effects of chemotherapy and drug resistance [3, 4]. Therefore, there is a great need for more effective therapeutic drugs to treat OSCC.

*Scutellaria baicalensis* Georgi (SBG) belongs to the Lamiaceae herb family with extensive biological and pharmacological activities, whose desiccative radix is an important component of the fundamental herb used in Traditional Chinese medicine (TCM) [5]. The active ingredients in SBG have important pharmacological effects including antioxidant, antibacterial, antitumor, and anti-inflammatory [5, 6]. Recently, SBG has been used to treat many diseases such as cancers, inflammation, hypertension, and immunologic disorders [7]. A wealth of evidence indicates that SBG may serve as essential source of novel therapeutic candidates for the treatment of OSCC due to its low side effects and remarkable activities [8–10]. However, the pharmacological mechanism of SBG in treating OSCC has not been fully elucidated for SBG is an herb containing various components.

**Figure 1:**
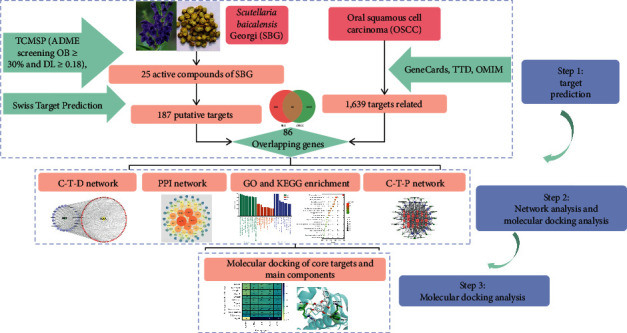
A flow diagram based on a cohesive integration strategy of network pharmacology and molecular docking.

TCM including SBG has the characteristics of “multicomponent, multitarget, and multipathway system.” [11] The recent development of network pharmacology has allowed the researcher to gain an understanding of the relationship of drugs with diseases and biological systems from a “Compound-Target-Disease” perspective [12]. Furthermore, molecular docking can predict the affinity of ligand and explain the mechanism of ligand action from the molecular level via exploring the interaction between ligand and receptor [13]. Kaplan–Meier Plotter online database was used to analyze the correlation between the core targets and the prognosis of OSCC further to verify the reliability of network pharmacology [14]. Utilizing these complementary techniques, we explored SBG targets and key signaling pathways that may provide potential strategies for treating OSCC. The flow diagram of the study is shown in Figure 1.

## 2. Materials and Methods

### 2.1. Acquisition of the Effective Components and Target of SBG

Traditional Chinese Medicine Systems Pharmacology (TCMSP, version: 2.3, https://tcmspw.com/tcmsp.php) database was used to collect the effective components of SBG [15]. Pharmacokinetic information retrieval filters absorption, distribution, metabolism, and excretion (ADME) were used to retrieve bioactive compounds for further analysis; we set oral bioavailability (OB) ≥30% and drug-likeness (DL) ≥0.18 as screening criteria [16]. After integrating with the results obtained from the above databases, the 2D molecular structures of potential active chemical constituents of SBG were downloaded from the PubChem (https://pubchem.ncbi.nlm.nih.gov/) [17] and were input into Swiss Target Prediction database (http://www.swisstargetprediction.ch/) [18]; click “Homo sapiens” to view all putative targets of the chemical constituents. The results are stored in TSV format for the following analysis.

### 2.2. Fishing for Putative Targets of OSCC

OSCC-related target genes were mined using GeneCards (https://www.genecards.org Version 5.0.0) [19], Online Mendelian Inheritance in Man (OMIM, https://omim.org/, updated March 21, 2020) [20], and Therapeutic Target Database (TTD, http://db.idrblab.org/ttd/, updated June 1, 2020) [21] using “oral squamous cell carcinoma” as the keywords.

### 2.3. Obtaining Common Targets for the Disease and Drug

To collect candidate targets for OSCC treatment, the presumed targets of SBG were mapped to OSCC-related targets to obtain common targets. The screened chemical targets and disease targets are imported into the Draw Venn Diagram (http://bioinformatics.psb.ugent.be/webtools/Venn/) platform for analysis [22], and then the common targets of both the disease and the drug obtained are used as the core targets for subsequent analysis.

### 2.4. Drawing the Component-Target-Disease Network

Cytoscape (https://www.cytoscape.org/, version:3.7.2) was used to visualize the component-target-disease network to reflect the complex links among active constituents, potential targets, and diseases [23]. The active components of SBG and common therapeutic targets were imported into Cytoscape software, and the main components were identified according to the topological parameters.

### 2.5. Protein-Protein Interaction (PPI) Network Map of Common Targets and Core Targets' Screening

Search Tool for the Retrieval of Interacting Genes/Proteins (STRING, http://stringdb.org/, version: 11.0) database was used to derive the PPI network [24]. Species was set as “Homo sapiens,” and the confidence score was set at the highest level (≥0.900); other parameters were set at default. Then, core targets were screened according to topological analysis. The file in TSV format was exported from the STRING database, and the downloaded results were uploaded to Cytoscape software for analysis.

### 2.6. Gene Ontology (GO) Functional Enrichment and Kyoto Encyclopedia of Genes and Genomes (KEGG) Pathway Analysis

Further analysis is required to reveal the specific mechanism of SBG in OSCC treatment. The common targets were uploaded into the Metascape system (http://metascape.org/, updated September 16, 2020) to complete GO functional analysis and KEGG pathways analysis [25], limited to “Homo sapiens,” and significance levels were set to *P* < 0.01.

### 2.7. Construction of the Component-Target-Pathway Network

Based on common targets of compound-disease and top predicted pathways, a “component-target-pathway” regulatory network was constructed using Cytoscape software [26].

### 2.8. Analysis of Core Target Genes and Overall Survival

The Kaplan–Meier Online website (http://kmplot.com/analysis/) and both the Cancer Genome Atlas (TCGA) and Gene Expression Omnibus (GEO) databases were used to analyze the relationship between the expression level of core targets and cancer prognosis [14, 27]. We used data from the Pan-cancer of the Kaplan–Meier Online website for “head-neck squamous cell carcinoma” to analyze the relationship between core target genes and OSCC patient prognosis. A total of 499 patients with OSCC were identified, and log-rank *P* < 0.05 was statistically significant.

### 2.9. Molecular Docking Analyses of the Main Active Components and Core Targets

A molecular docking tool (Systems Dock) was used to determine if the main components of SBG have the binding capacity with core targets. We selected core targets in the PPI and Component-Targets-Pathways networks as potential receptors based on the degree value. The PubChem database was used to obtain 3D structures of the main active components of SBG, and the crystal structures of core target genes were directly obtained from the RCSB PDB database (https://www.rcsb.org/) [26]. AutoDock Tools 1.5.6 software was used to process the above protein receptors and ligands routinely and save them as a PDBQT file. Auto grid module was used to obtain the active docking site, run the program for molecular docking, and receive relevant binding energy (affinity). Autodock Vina 1.1.2 was used to perform bioinformatics studies and molecular docking. PyMOL 1.7.x software-based visualization analyses showed the conformation with the best affinity. The main active components of SBG were compared with 5-fluorouracil and cisplatin, which are the first-line treatment of OSCC [4]. Finally, a heat map was made based on the strongest affinity of core targets and main ingredients of SBG.

## 3. Results

### 3.1. Potential Targets of SBG

A total of 25 active chemical ingredients of SBG were obtained through the screening criteria properties (OB ≥30% and DL ≥0.18) based on TCMSP (Table 1 and Supplementary File, Table S1). The 2D SDF structure of these 25 compounds was uploaded to search for the potential therapeutic targets from the Swiss Target Prediction database, and 2,102 component-related targets were predicted (Supplementary File, Table S2). In total, there were 187 potential targets of SBG after duplicates were removed.

### 3.2. Potential Targets of OSCC

GeneCards, OMIM, and TTD were used to collect OSCC-related targets. By using the keywords “oral squamous cell carcinoma” in sequence, 4,586 OSCC-related targets were obtained from GeneCards, and then we chose 1,147 targets two times greater than the median of relevance score from GeneCards, 469 OSCC-related genes from OMIM, and 1 OSCC-related target from TTD, respectively (Supplementary File, Table S3). As a result, a total of 1,639 targets associated with OSCC pathogenesis were obtained after duplicates had been deleted.

### 3.3. Converting Putative Targets of SBG against OSCC into Network and Analysis

**Figure 2:**
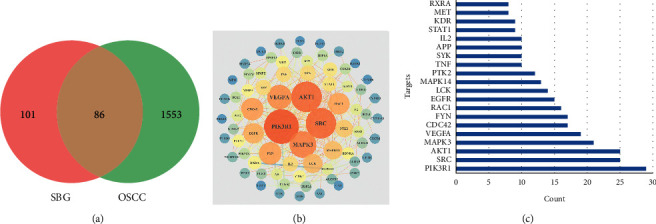
Construction of the PPI network and identification of the core targets. (a) Venn diagram of the intersection of 187 targets of SBG and 1,639 targets of OSCC. (b) PPI network of potential core targets related to SBG and OSCC. The nodes in lighter color and a more significant size represented a higher degree. The edges in lighter color and a wider size presented a higher combined score. (c) The top 30 core targets in the PPI network (the *y*-axis shows the top 20 significant targets, and the *x*-axis shows the count of interconnected targets).

Based on the above results, we identified 86 common targets of SBG into pathological targets by the intersection of 187 putative targets of SBG and 1,639 OSCC-related targets. The 86 common targets served as the putative therapeutic targets of SBG against OSCC (Figure 2(a), Supplementary File, Table S4). Furthermore, 86 potential genes were uploaded to the STRING database for analysis. There were a total of 86 nodes, 239 edges, and 5.56 average node degrees. Further analysis was done using the Cytoscape software, and the PPI network was visualized (Figure 2(b), Supplementary File, Table S5). The top 20 core targets were then selected (Figure 2(c)). From the PPI network and the bar graph (Figure 2(c)), we found that the leading core targets are phosphoinositide-3-kinase regulatory subunit 1 (PIK3R1), SRC proto-oncogene (SRC), AKT serine/threonine kinase 1 (AKT1), mitogen-activated protein kinase 3 (MAPK3), and vascular endothelial growth factor A (VEGFA) (Table 2); these were considered as the core targets of SBG and may play a pivotal role in the development of OSCC.

### 3.4. Visualizing the Compound-Target-Disease Network

**Figure 3:**
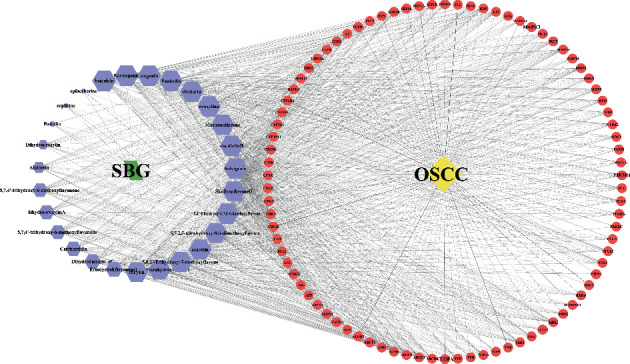
Construction of the compound-target-disease network (the blue hexagon nodes represent active compounds of SBG, and pink ellipse nodes represent potential therapeutic targets. Nodes' sizes are proportional to their degree).

To build a compound-target-disease network diagram, we imported the 25 active ingredients of SBG and 86 common targets into the Cytoscape software resulting in a total of 139 nodes and 546 connections in this network (Figure 3). According to topological analysis (Supplementary File, Table S6), we found that the leading hub compounds are baicalein, norwogonin, wogonin, oroxylin-A, salvigenin, moslosooflavone, rivularin, skullcapflavone II, and viscidulin II, which are also the core components in SBG with anti-OSCC effect.

### 3.5. GO and KEGG Pathway Enrichment Analysis

**Figure 4:**
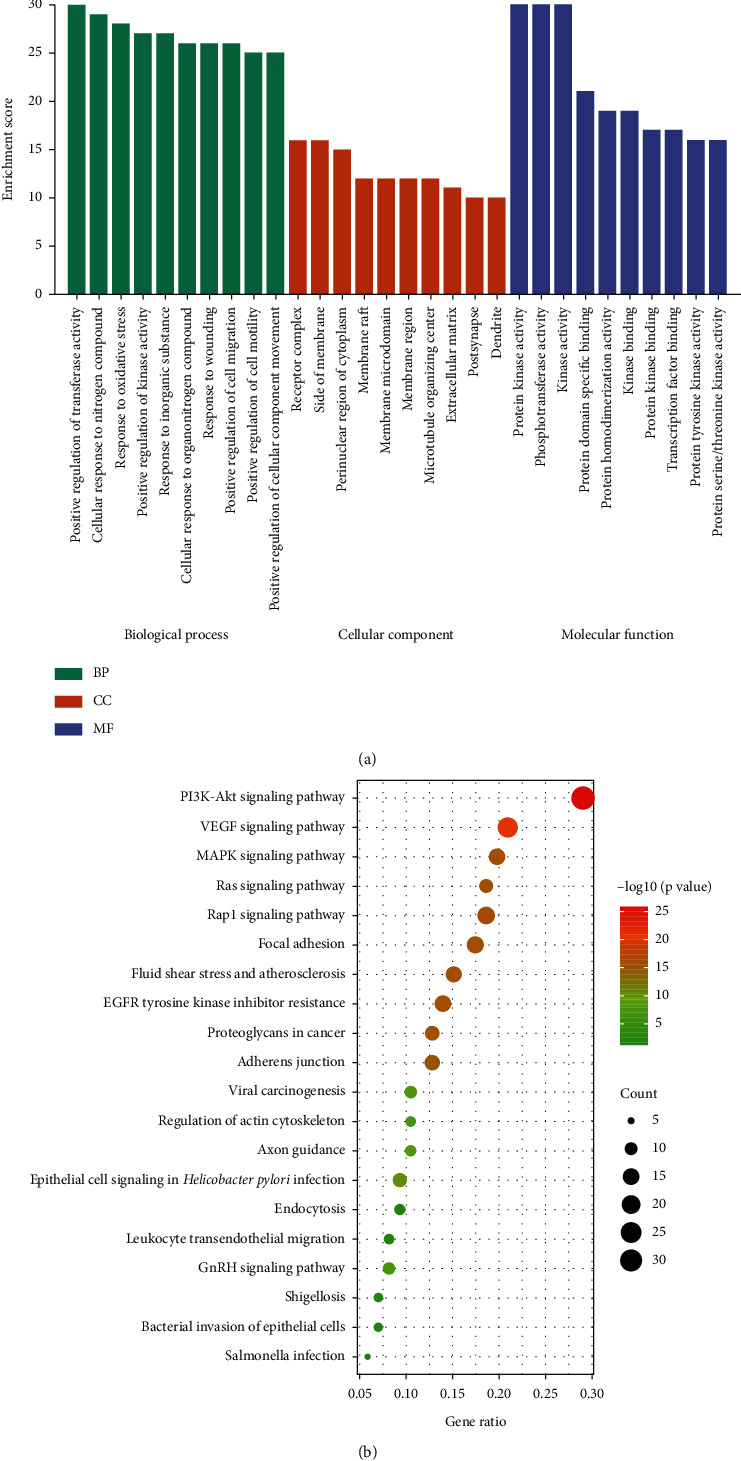
GO enrichment and KEGG pathway enrichment analysis of the 86 potential therapeutic targets. (a) GO enrichment analysis: the top 10 biological processes, molecular functions, and cell components, *P* < 0.01. (b) Bubble chart of the top 20 KEGG pathways, *P* < 0.01.

We imported the 86 potential therapeutic targets into the Metascape system for GO and KEGG analysis to further elucidate the mechanism of SBG against OSCC. Biological processes (BP), molecular functions (MF), and cellular components (CC) related to the GO results revealed the functions of these potential targets. A total of 1,846 significant enriched GO entries were obtained from Metascape, including 1,653 BP, 115 MF, and 78 CC entries based on *P* < 0.01 (Supplementary File, Table S7). Out of 1,653 BP GO terms, the most enriched BP terms were positive regulation of transferase activity, cellular response to nitrogen compounds, and response to oxidative stress (Figure 4(a)). And out of 115 MF GO terms, the most important terms associated SBG treatment were protein kinase activity, phosphotransferase activity, and kinase activity (Figure 4(a)). Among 78 CC GO terms associated SBG treatment, receptor complex, side of membrane, and perinuclear region of the cytoplasm were included in the top 10 entries (Figure 4(a)). A total of 141 pathways were enriched based on *P* < 0.01 (Supplementary File, Table S8); the pathways with the highest enrichment level included PI3K-Akt, EGFR, and MAPK signaling, primarily involved in the immune-related and inflammatory-related function. Based on *P* values and counts of hit genes, the top 10 GO entries, and top 20 KEGG pathways were chosen (Figures 4(a) and 4(b)).

### 3.6. The Compound-Target-Pathway Network of SBG for OSCC

**Figure 5:**
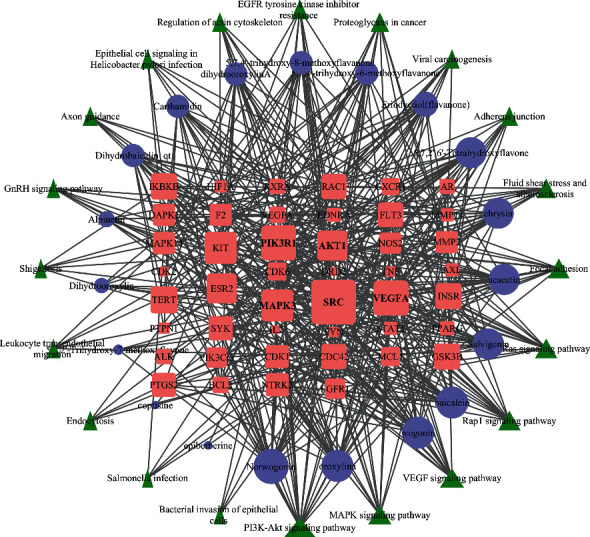
The network of “compound-target-pathway” of SBG for OSCC (the pink nodes represent the target of SBG for OSCC, the purple nodes represent the drug composition, and the green nodes represent the signaling pathway for SBG against OSCC).

The compound-target-pathway network analysis is shown in Figure 5, and the specific data are given in Table 3. The network relationship between the top 20 pathways and their regulated target genes is shown in the diagram. According to topological analysis, PIK3R1, SRC, AKT1, MAPK3, and VEGFA had the highest volume in genes and thus may be critical in the SBG treatment of OSCC. A relatively high volume consistent on the compound-target-disease network was also observed among baicalein, norwogonin, wogonin, oroxylin-A, and salvigenin.

### 3.7. Analysis of the Core Target Genes on Median Survival of OSCC

**Figure 6:**
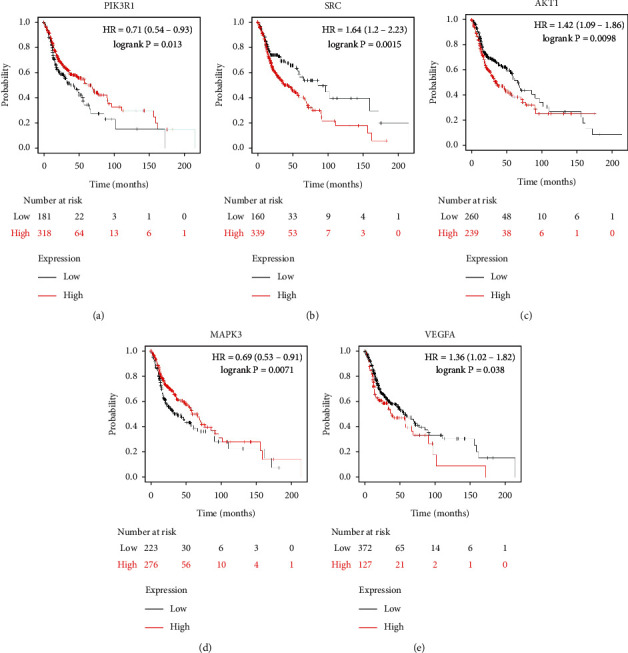
Survival curve analysis of five core target genes and median survival in OSCC patients. The five core target genes are (a) PIK3R1, (b) SRC, (c) AKT1, (d) MAPK3, and (e) VEGFA.

We used the Kaplan–Meier Plotter online database to analyze the correlation between five identified genes (PIK3R1, SRC, AKT1, MAPK3, and VEGFA) and OSCC prognosis. Correlation analysis showed that the expressions of PIK3R1, SRC, AKT1, MAPK3, and VEGFA were all related to the median survival time of OSCC patients (*P* < 0.05). The median survival time of the patients with high expression of PIK3R1 and MAPK3 was better than that of the low expression group, and increased expression of SRC, AKT1, and VEGFA was worse than low SRC, AKT1, and VEGFA expression as shown in Figure 6. The results suggest that PIK3R1, SRC, AKT1, MAPK3, and VEGFA as core target genes of SBG were significant associations with survival time in patients with OSCC.

### 3.8. Molecular Docking Analysis

**Figure 7:**
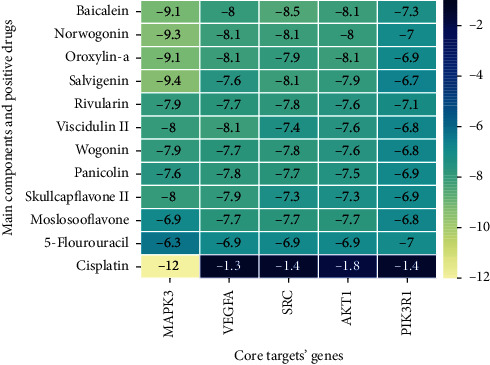
The binding energy of the main active components of SBG and core target genes.

**Figure 8:**
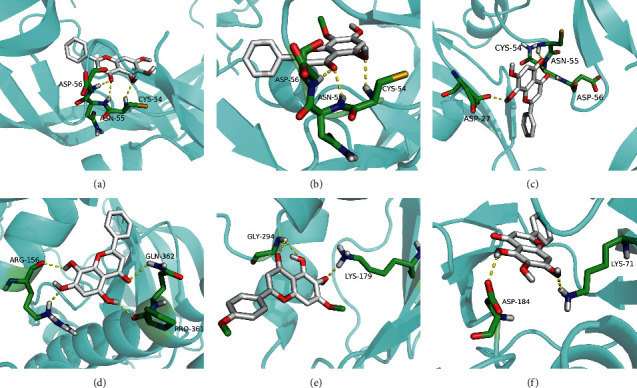
The result of molecular docking of the main components and core targets. (a) Baicalein-VEGFA. (b) Wogonin-VEGFA. (c) Norwogonin-VEGFA. (d) Norwogonin-SRC. (e) Salvigenin-AKT1. (f) Baicalein-MAPK3.

Molecular docking analysis provided a visual interaction between the chemical components of SBG and OSCC-related genes. Cisplatin and 5-fluorouracil are used as positive drugs for molecular docking. The smaller the binding energy, the stronger the binding force between the component and target. Affinity <−4.25 kcal/mol means that ligands and receptors have the possibility of combination, affinity <−5.00 kcal/mol indicates good binding strength, and affinity <−7.00 kcal/mol suggests satisfactory binding strength [28]. As previously described, 60 pairs resulted from molecular docking analysis (Supplementary File, Table S9). From the above results, as compared to 5-fluorouracil and cisplatin, the main components and core targets have better binding activity (affinity <−7.00 kcal/mol). Baicalein has four hydrogen bonds with the amino acid residues bound to VEGFA and formed a stable complex (Figure 7(a)). The specific molecular docking results are shown in Table 4, and a visual result is shown in Figure 7. We placed the best pairs of compounds-targets into PyMOL software for visualization, and the specific protein-ligand interactions of docking are shown in Figure 8.

## 4. Discussion

OSCC is an inadequately treated cancer that affects the quality of life of patients [29]. Surgery is primarily used for early treatment, and combination therapy (chemotherapy and radiotherapy) is commonly used to prevent recurrence and improve efficacy, but there are side effects. Therefore, it is essential to develop new drugs to reduce the morbidity and mortality of OSCC. TCM has been widely accepted as a mainstream form of complementary and alternative therapy beneficial to cancer patients because it inhibits and kills tumor cells and can reduce radiotherapy and chemotherapy's adverse effects [11]. TCM treatment also improves patients' quality of life and immunity, reduces clinical symptoms, and prolongs survival time [30]. SBG is one of the TCM that has been reported to possess anticancer, antioxidant, anti-inflammatory, antiviral, and antibacterial activities [31].

Our data showed 25 active compounds in SBG that act on 86 different targets associated with OSCC. Five flavonoid compounds, baicalein, wogonin, oroxylin-A, salvigenin, and norwogonin, are highly connected potential therapeutic targets and pathways of SBG against OSCC which can be defined as decisive compounds in OSCC according to the topological parameters. The five compounds play protective roles against tumor cell development, and dietary intake of flavonoids can significantly reduce the risk of cancer and inflammation [32]. The previous study has shown that baicalein can induce cell cycle arrest and apoptosis to treat and prevent cancer without adverse side effects [33]. Gao et al. have demonstrated that baicalein suppresses OSCC cells' growth through Sp1/NF-*κ*B dependent mechanism [8]. Wogonin has been thoroughly studied for its anticancer, antiviral, and antioxidant activity [34]. Wogonin's tumor inhibitory properties may be associated with targeting PI3K-Akt and MAPK pathways, inhibiting the cell cycle arrest and overcoming drug resistance [35]. Oroxylin-A possesses pharmacological effects of anticancer, anti-inflammatory, neuroprotective, and anticoagulant activities [36]. Slavigenin is also a potent anticancer agent active against various human cancer cell lines [37], and it has been found to have antiproliferative, anti-inflammatory, and cytotoxic effects [38]. Hypoxia induces EMT and increases migration and metastasis in OSCC [39, 40]. And previous studies have articulated that norwogonin can attenuate hypoxia-induced oxidative stress and apoptosis [41]. Therefore, these main components of SBG play an essential role in anti-OSCC, which is consistent with previous studies.

By integrating network analyses, we revealed that SBG acts on multiple targets using multiple signaling pathways, mainly PIK3R1, SRC, AKT1, MAPK3, and VEGFA, potentially core target genes that could be pivotal in the development of OSCC. PIK3R1 is the predominant regulatory isoform of PI3K, a tumor-suppressor gene in OSCC, regulating cancer cell proliferation [42, 43]. The expression and activity of SRC are associated with advanced malignancy and poor prognosis in a variety of human cancers. SRC plays a vital role in cell proliferation, invasion, movement, and signal transduction [44]. AKT1 plays a key oncogene role in human OSCC cells and may be a critical target for novel therapy development [45]. As a member of the mitogen-activated protein kinase (MAPK) family, the abnormal expression of MAPK3 is related to the invasion and metastasis of various tumor cells [46]. VEGFA is the most important proangiogenic factor and plays a crucial role in angiogenesis. Activated VEGFA can promote endothelial cell proliferation and migration and lead to new blood vessels' formation [47]. The above genes are associated with inflammation, cell proliferation, invasion, and angiogenesis, which are closely related to the occurrence and development of OSCC.

In our studies, KEGG enrichment analysis showed that 86 common targets were mainly related to the PI3K-Akt, VEGF, and MAPK signaling pathways. SBG may treat OSCC via the PI3K-Akt signaling pathway, which is an essential signaling pathway in regulating the cell cycle and can be activated by multiple types of stimulation or toxic injury. It plays a vital role in the chronic inflammatory response of OSCC [48]. As a downstream molecule of the P13K-Akt signal transduction pathway, mTOR plays a crucial role in tumorigenesis, invasion, metastasis, and angiogenesis. Phosphorylated mTOR (P-MTOR) overexpression is associated with poor prognosis in OSCC [49]. It was reported that 53% of tongue squamous cell carcinoma had p-MTOR overexpression [50]. The PI3K-Akt signaling pathway is altered in OSCC [51]. Invasion and migration can be suppressed in OSCC by inhibition of the PI3K-Akt signaling pathway [52]. Baicalein and wogonin induce apoptosis in many cancer cells by modulating the PI3K-Akt pathway [53, 54]. Angiogenesis is an essential feature of the progression of OSCC and VEGF plays critical roles in OSCC tumor angiogenesis and metastasis [55]. Meanwhile, VEGF-Flt-1 signaling can induce osteoclastogenesis in OSCC [56]. Previous studies have shown that the MAPK signaling pathway is involved in the development of OSCC, and TNF-*α* regulates EMT through the MAPK signaling pathway to promote the invasion and metastasis of OSCC [57, 58]. Baicalein plays an anti-inflammatory role by inhibiting the MAPK signaling pathway in pancreatitis, colon cancer, and thyroid cancer [59–61]. Thus, our data supported that SBG exerts its anti-OSCC effect by inhibiting cell proliferation, reducing inflammation via multiple pathways.

The GO of the BP was analyzed in our studies further to confirm the relationship between potential targets and OSCC. According to our analysis, the most enriched BP were positive regulation of transferase activity, cellular response to nitrogen compounds, and response to oxidative stress. Nitrogen acquisition and utilization have essential effects on cancer and immunity [62]. Oxidative stress and its induced oxidative damage are important factors in OSCC formation and development [63, 64], which provided another insight into the mechanism of SBG in the treatment of OSCC.

In this study, the data of the core target genes on median survival of OSCC patients suggested that PIK3R1, SRC, AKT1, MAPK3, and VEGFA are potential therapeutic targets of SBG; there was a correlation with the survival time of OSCC patients. PIK3R1, SRC, AKT1, MAPK3, and VEGFA are common therapeutic targets for OSCC closely related to the survival and prognosis of OSCC patients [31, 53, 65, 66], which further supported the potential of SBG in the treatment of OSCC.

Molecular docking is the most common approach for evaluating component-target interactions. Baicalein, norwogonin, and oroxylin-A were the most stable active ingredient in core targets binding, and their docking scores were all<−8.00 kcal/mol. Wogonin, salvigenin, rivularin, skullcapflavone II, moslosooflavone, panicolin, and viscidulin showed a strong association with five core targets' docking scores being all<−7.00 kcal/mol. Nevertheless, compared to 5-fluorouracil and cisplatin, main components of SBG can bind to core targets of OSCC more stably on molecular docking anastomosis. These results suggest that an interaction does not mediate SBG treatment for OSCC between a single compound in SBG and a target protein in OSCC, but among multiple compounds in SBG and numerous target proteins in OSCC. Our data revealed that the effectiveness and mechanisms of SBG treatment on OSCC are complicated, which involved multicomponent interactions.

## 5. Conclusions

In the present study, network pharmacology identified 25 main active compounds in SBG and 86 genes targeted by OSCC. Five core targets (PIK3R1, SRC, AKT1, MAPK3, and VEGFA) from complex networks through the integration of network pharmacology were predicted to be critical targets of SBG in treating OSCC. Besides, we found that the PI3K-Akt signaling pathway may occupy core status in the anti-OSCC system. Results from molecular docking further revealed that several SBG compounds exhibited an excellent affinity for their core targets. These results elucidate the potentially effective mechanisms of SBG to treat OSCC on multiple targets and multiple pathways, which also shed a light on the development of the new drug for OSCC.

## Figures and Tables

**Table 1:** Basic information of 25 potential active components in SBG.

Number	Mol ID	Molecule name	OB (%)	DL	Molecular structure
1	MOL000173	Wogonin	30.68	0.23	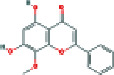
2	MOL000552	5,2′-Dihydroxy-6,7,8-trimethoxyflavone	31.71	0.35	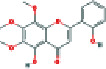
3	MOL002714	Baicalein	33.52	0.21	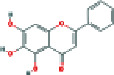
4	MOL002909	5,7,2,5-Tetrahydroxy-8,6-dimethoxyflavone	33.82	0.45	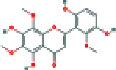
5	MOL001689	Acacetin	34.97	0.24	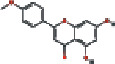
6	MOL012246	5,7,4′-Trihydroxy-8-methoxyflavanone	74.24	0.26	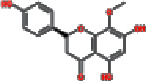
7	MOL012245	5,7,4′-Trihydroxy-6-methoxyflavanone	36.63	0.27	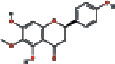
8	MOL002908	5,8,2′-Trihydroxy-7-methoxyflavone	37.01	0.27	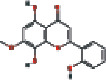
9	MOL002925	5,7,2′,6′-Tetrahydroxyflavone	37.01	0.24	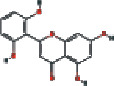
10	MOL012266	Rivularin	37.94	0.37	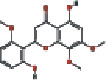
11	MOL002926	Dihydrooroxylin A	38.72	0.23	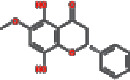
12	MOL000525	Norwogonin	39.4	0.21	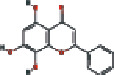
13	MOL002913	Dihydrobaicalin_qt	40.04	0.21	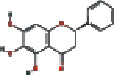
14	MOL002910	Carthamidin	41.15	0.24	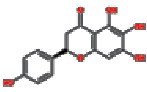
15	MOL002914	Eriodyctiol (flavanone)	41.35	0.24	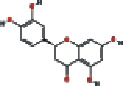
16	MOL002928	Oroxylin-A	41.37	0.23	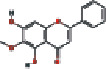
17	MOL008206	Moslosooflavone	44.09	0.25	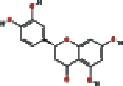
18	MOL002917	Viscidulin II	45.05	0.33	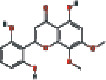
19	MOL002915	Salvigenin	49.07	0.33	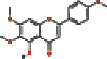
20	MOL000228	Alpinetin	55.23	0.20	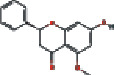
21	MOL002927	Skullcapflavone II	69.51	0.44	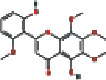
22	MOL002932	Panicolin	76.26	0.29	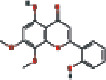
23	MOL001458	Coptisine	30.67	0.86	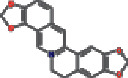
24	MOL002897	Epiberberine	43.09	0.78	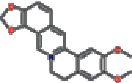
25	MOL002937	Dihydrooroxylin	66.06	0.23	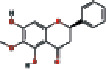

**Table 2:** Basic information about the 5 core targets.

Number	Uniport ID	Protein name	Gene name	Degree	Closeness
1	P27986	Phosphoinositide-3-kinase regulatory subunit 1	PIK3R1	29	0.51079137
2	P12930	SRC proto-oncogene	SRC	25	0.50714286
3	P31750	AKT serine/threonine kinase 1	AKT1	25	0.51079137
4	P27361	Mitogen-activated protein kinase 3	MAPK3	21	0.48965517
5	P15692	Vascular endothelial growth factor A	VEGFA	19	0.5035461

**Table 3:** KEGG pathway enrichment results (top 20).

Serial number	Pathway	Count	Gene ratio	*P* value
hsa04151	PI3K-Akt signaling pathway	25	0.291	1.63609E-08
hsa04370	VEGF signaling pathway	18	0.209	7.83713E-07
hsa04010	MAPK signaling pathway	16	0.186	8.23084E-06
hsa04014	Ras signaling pathway	12	0.140	8.40212E-06
hsa04015	Rap1 signaling pathway	11	0.128	1.49134E-05
hsa04510	Focal adhesion	16	0.186	1.51741E-05
hsa05418	Fluid shear stress and atherosclerosis	17	0.198	1.6281E-05
hsa01521	EGFR tyrosine kinase inhibitor resistance	15	0.174	1.74787E-05
hsa05205	Proteoglycans in cancer	13	0.151	2.97748E-05
hsa04520	Adherens junction	11	0.128	3.22947E-05
hsa05203	Viral carcinogenesis	8	0.093	0.000800529
hsa04810	Regulation of actin cytoskeleton	9	0.105	0.003872469
hsa04360	Axon guidance	9	0.105	0.004695093
hsa05120	Epithelial cell signaling in helicobacter pylori infection	7	0.081	0.004967716
hsa04912	GnRH signaling pathway	9	0.105	0.006207927
hsa05131	Shigellosis	6	0.070	0.006899158
hsa04670	Leukocyte transendothelial migration	7	0.081	0.007712769
hsa05100	Bacterial invasion of epithelial cells	6	0.070	0.01033839
hsa04144	Endocytosis	8	0.093	0.019022329
hsa05132	Salmonella infection	5	0.058	0.032457397

**Table 4:** The binding energy of top 10 compounds of SBG with OSCC-related core targets.

	Molecular name	CAS ID	Core gene docking score (kcal/mol)				
			MAPK3 PDB ID (4QTB)	VEGFA PDB ID (3QTK)	SRC PDB ID (1FMK)	AKT1 PDB ID (4GV1)	PIK3R1 PDB ID (6D85)
Core components	Baicalein	491-67-8	−9.1	−8	−8.5	−8.1	−7.3
	Norwogonin	4443-09-8	−9.3	−8.1	−8.1	−8	−7
	Oroxylin-A	480-11-5	−9.1	−8.1	−7.9	−8.1	−6.9
	Salvigenin	19103-54-9	−9.4	−7.6	−8.1	−7.9	−6.7
	Rivularin	70028-59-0	−7.9	−7.7	−7.8	−7.6	−7.1
	Viscidulin II	92519-93-2	−8.0	−8.1	−7.4	−7.6	−6.8
	Wogonin	632-85-9	−7.9	−7.7	−7.8	−7.6	−6.8
	Panicolin	41060-16-6	−7.6	−7.8	−7.7	−7.5	−6.9
	Skullcapflavone II	55084-08-7	−8.0	−7.9	−7.3	−7.3	−6.9
	Moslosooflavone	3570-62-5	−6.9	−7.7	−7.7	−7.7	−6.8

Positive drugs	5-Flourouracil	51-21-8	−6.3	−6.9	−6.9	−6.9	
	Cisplatin	15663-27-1	−12.3	−1.3	−1.4	−1.8	−1.4

## Data Availability

All the data are available from the first author upon reasonable request. The data used to support the findings of this study are included within the supplementary information files.

## Abbreviations

OSCC: Oral squamous cell carcinoma
SBG: *Scutellaria baicalensis* Georgi
TCMSP: Traditional Chinese Medicine Systems Pharmacology Database
OMIM: Online Mendelian Inheritance in Man
TTD: Therapeutic Target Database
PPI: Protein-protein interaction
GO: Gene Ontology
KEGG: Kyoto Encyclopedia of Genes and Genomes
CC: Cellular components
MF: Molecular functions
BP: Biological processes
TCM: Traditional Chinese medicine
ADME: Absorption, distribution, metabolism, and excretion
OB: Oral bioavailability
DL: Drug-likeness
STRING: Search Tool for the Retrieval of Interacting Genes/Proteins
TCGA: The Cancer Genome Atlas
GEO: Gene Expression Omnibus
PIK3R1: Phosphoinositide-3-kinase regulatory subunit 1
SRC: SRC proto-oncogene
AKT1: AKT serine/threonine kinase 1
MAPK3: Mitogen-activated protein kinase 3
VEGFA: Vascular endothelial growth factor A.

## Supplementary Materials

Supplementary Materials Table S1: detailed information of active compounds in SBG. Table S2: target gene-related active compounds of SBG. Table S3: list of OSCC-related genes in the GeneCards database, OMIM, and TTD. Table S4: the putative targets of SBG against OSCC. Table S5: topological analysis of the PPI network. Table S6: topological analysis of the compound-target-disease network. Table S7: the GO enrichment analysis for intersection targets between compound and OSCC-related targets. Table S8: the enriched KEGG pathways for intersection targets between compound and AD-related targets. Table S9: the results of molecular docking.
