# Supplementary material for: Exploring the Mechanism of Scutellaria baicalensis Georgi Efficacy against Oral Squamous Cell Carcinoma Based on Network Pharmacology and Molecular Docking Analysis
Source: Evid Based Complement Alternat Med. 2021 Jul 13;2021:5597586. doi: 10.1155/2021/5597586 (PMC8292061; doi:10.1155/2021/5597586)
Supplement: Supplementary Materials — Table S1: detailed information of active compounds in SBG. Table S2: target gene-related active compounds of SBG. Table S3: list of OSCC-related genes in the GeneCards database, OMIM, and TTD. Table S4: the putative targets of SBG against OSCC. Table S5: topological analysis of the PPI network. Table S6: topological analysis of the compound-target-disease network. Table S7: the GO enrichment analysis for intersection targets between compound and OSCC-related targets. Table S8: the enriched KEGG pathways for intersection targets between compound and AD-related targets. Table S9: the results of molecular docking. [file 5597586.f1.zip › 5597586.f1/Supplementary Description.docx]

## Supplementary Description:

Table S1. Detailed information of active compounds in SBG. Table S2. Target genes related active compounds of SBG. Table S3. List of OSCC related genes in the GeneCard database, OMIM and TTD. Table S4. The putative targets of SBG against OSCC. Table S5. Topological analysis of the PPI network. Table S6. Topological analysis of the Compound-Target-Disease network. Table S7. The GO enrichment analysis for intersection targets between compound and OSCC related targets. Table S8. The enriched KEGG pathways for intersection targets between compound and AD related targets. Table S9. The results of molecular docking. *(Supplementary Materials)*
