# Supplementary material for: Exploring the Mechanism of Scutellaria baicalensis Georgi Efficacy against Oral Squamous Cell Carcinoma Based on Network Pharmacology and Molecular Docking Analysis
Source: Evid Based Complement Alternat Med. 2021 Jul 13;2021:5597586. doi: 10.1155/2021/5597586 (PMC8292061; doi:10.1155/2021/5597586)
Supplement: Supplementary Materials — Table S1: detailed information of active compounds in SBG. Table S2: target gene-related active compounds of SBG. Table S3: list of OSCC-related genes in the GeneCards database, OMIM, and TTD. Table S4: the putative targets of SBG against OSCC. Table S5: topological analysis of the PPI network. Table S6: topological analysis of the compound-target-disease network. Table S7: the GO enrichment analysis for intersection targets between compound and OSCC-related targets. Table S8: the enriched KEGG pathways for intersection targets between compound and AD-related targets. Table S9: the results of molecular docking. [file 5597586.f1.zip › 5597586.f1/Supplementary File 1. Detailed information of active compounds in SBG.pdf]

Table S1. Detailed information of active compounds (OB ≥ 30% and DL ≥ 0.18) in SBG.

No.	Herb	Source	Mol ID	Molecule Name	OB(%)	DL
1	SBG	TCMPS	MOL000173	wogonin	30.68	0.23
2	SBG	TCMPS	MOL000552	5,2'-Dihydroxy-6,7,8-trimethoxyflavone	31.71	0.35
3	SBG	TCMPS	MOL002714	baicalein	33.52	0.21
4	SBG	TCMPS	MOL002909	5,7,2,5-tetrahydroxy-8,6-dimethoxyflavone	33.82	0.45
5	SBG	TCMPS	MOL001689	acacetin	34.97	0.24
6	SBG	TCMPS	MOL012246	5,7,4'-trihydroxy-8-methoxyflavanone	74.24	0.26
7	SBG	TCMPS	MOL012245	5,7,4'-trihydroxy-6-methoxyflavanone	36.63	0.27
8	SBG	TCMPS	MOL002908	5,8,2'-Trihydroxy-7-methoxyflavone	37.01	0.27
9	SBG	TCMPS	MOL002925	5,7,2',6'-Tetrahydroxyflavone	37.01	0.24
10	SBG	TCMPS	MOL012266	rivularin	37.94	0.37
11	SBG	TCMPS	MOL002926	dihydrooroxylin A	38.72	0.23
12	SBG	TCMPS	MOL000525	Norwogonin	39.4	0.21
13	SBG	TCMPS	MOL002913	Dihydrobaicalin_qt	40.04	0.21
14	SBG	TCMPS	MOL002910	Carthamidin	41.15	0.24
15	SBG	TCMPS	MOL002914	Eriodyctiol (flavanone)	41.35	0.24
16	SBG	TCMPS	MOL002928	oroxylin a	41.37	0.23
17	SBG	TCMPS	MOL008206	Moslosooflavone	44.09	0.25
18	SBG	TCMPS	MOL002917	viscidulin II	45.05	0.33
19	SBG	TCMPS	MOL002915	Salvigenin	49.07	0.33
20	SBG	TCMPS	MOL000228	Alpinetin	55.23	0.2
21	SBG	TCMPS	MOL002927	Skullcapflavone II	69.51	0.44
22	SBG	TCMPS	MOL002932	Panicolin	76.26	0.29
23	SBG	TCMPS	MOL001458	coptisine	30.67	0.86
24	SBG	TCMPS	MOL002897	epiberberine	43.09	0.78
25	SBG	TCMPS	MOL002937	Dihydrooroxylin	66.06	0.23
