# Supplementary material for: Exploring the Mechanism of Scutellaria baicalensis Georgi Efficacy against Oral Squamous Cell Carcinoma Based on Network Pharmacology and Molecular Docking Analysis
Source: Evid Based Complement Alternat Med. 2021 Jul 13;2021:5597586. doi: 10.1155/2021/5597586 (PMC8292061; doi:10.1155/2021/5597586)
Supplement: Supplementary Materials — Table S1: detailed information of active compounds in SBG. Table S2: target gene-related active compounds of SBG. Table S3: list of OSCC-related genes in the GeneCards database, OMIM, and TTD. Table S4: the putative targets of SBG against OSCC. Table S5: topological analysis of the PPI network. Table S6: topological analysis of the compound-target-disease network. Table S7: the GO enrichment analysis for intersection targets between compound and OSCC-related targets. Table S8: the enriched KEGG pathways for intersection targets between compound and AD-related targets. Table S9: the results of molecular docking. [file 5597586.f1.zip › 5597586.f1/Supplementary File 2. Target genes related active compounds of SBG.pdf]

Table S2. All genes related active compounds through ADME screening based on SwissTarget Prediction database.

MOL ID	MolName	Target	Commmon name	Probilitity
MOL001689	acacetin	Cytochrome P450 1B1	CYP1B1	0.320218721
MOL001689	acacetin	Tankyrase-2	TNKS2	0.213125923
MOL001689	acacetin	Tankyrase-1	TNKS	0.213125923
MOL001689	acacetin	Stem cell growth factor receptor	KIT	0.180252494
MOL001689	acacetin	Delta opioid receptor	OPRD1	0.180252494
MOL001689	acacetin	NADPH oxidase 4	NOX4	0.171978589
MOL001689	acacetin	Aldose reductase (by homology)	AKR1B1	0.171978589
MOL001689	acacetin	Cyclin-dependent kinase 5/CDK5 activator 1	CDK5R1	0.171978589
MOL001689	acacetin	Xanthine dehydrogenase	XDH	0.171978589
MOL001689	acacetin	Monoamine oxidase A	MAOA	0.171978589
MOL001689	acacetin	Tyrosine-protein kinase receptor FLT3	FLT3	0.171978589
MOL001689	acacetin	Cytochrome P450 19A1	CYP19A1	0.171978589
MOL001689	acacetin	Estrogen receptor alpha	ESR1	0.171978589
MOL001689	acacetin	Cyclin-dependent kinase 1/cyclin B	CCNB3	0.171978589
MOL001689	acacetin	Acetylcholinesterase	ACHE	0.171978589
MOL001689	acacetin	Adenosine A1 receptor (by homology)	ADORA1	0.171978589
MOL001689	acacetin	Cyclooxygenase-2	PTGS2	0.171978589
MOL001689	acacetin	Estrogen receptor beta	ESR2	0.171978589
MOL001689	acacetin	Cyclin-dependent kinase 6	CDK6	0.171978589
MOL001689	acacetin	Adenosine A2a receptor (by homology)	ADORA2A	0.171978589
MOL001689	acacetin	Tyrosine-protein kinase SYK	SYK	0.171978589
MOL001689	acacetin	Glycogen synthase kinase-3 beta	GSK3B	0.171978589
MOL001689	acacetin	Multidrug resistance-associated protein 1	ABCC1	0.171978589
MOL001689	acacetin	Estradiol 17-beta-dehydrogenase 1	HSD17B1	0.171978589
MOL001689	acacetin	Transthyretin	TTR	0.171978589
MOL001689	acacetin	Casein kinase II alpha	CSNK2A1	0.171978589
MOL001689	acacetin	Cystic fibrosis transmembrane conductance regulator	CFTR	0.171978589
MOL001689	acacetin	ATP-binding cassette sub-family G member 2	ABCG2	0.171978589
MOL001689	acacetin	Aldo-keto reductase family 1 member B10	AKR1B10	0.171978589
MOL001689	acacetin	Serine/threonine-protein kinase PIM1	PIM1	0.163737226
MOL001689	acacetin	P-glycoprotein 1	ABCB1	0.163737226
MOL001689	acacetin	Adenosine A3 receptor	ADORA3	0.139061947
MOL001689	acacetin	Receptor-type tyrosine-protein phosphatase S	PTPRS	0.139061947
MOL001689	acacetin	AMY1C	AMY1A	0.139061947
MOL001689	acacetin	G protein-coupled receptor kinase 6	GRK6	0.139061947
MOL001689	acacetin	Androgen Receptor	AR	0.139061947
MOL001689	acacetin	Carbonic anhydrase II	CA2	0.139061947
MOL001689	acacetin	Carbonic anhydrase VII	CA7	0.139061947

MOL001689	acacetin	Carbonic anhydrase I	CA1	0.139061947
MOL001689	acacetin	Carbonic anhydrase XII	CA12	0.139061947
MOL001689	acacetin	Carbonic anhydrase IX	CA9	0.139061947
MOL001689	acacetin	Carbonic anhydrase IV	CA4	0.139061947
MOL001689	acacetin	Carbonyl reductase [NADPH] 1	CBR1	0.139061947
MOL001689	acacetin	Plasminogen	PLG	0.139061947
MOL001689	acacetin	Arachidonate 5-lipoxygenase	ALOX5	0.122581769
MOL001689	acacetin	Glyoxalase I	GLO1	0.122581769
MOL001689	acacetin	Beta amyloid A4 protein	APP	0.122581769
MOL001689	acacetin	Poly [ADP-ribose] polymerase-1	PARP1	0.122581769
MOL001689	acacetin	Matrix metalloproteinase 9	MMP9	0.122581769
MOL001689	acacetin	Matrix metalloproteinase 2	MMP2	0.122581769
MOL001689	acacetin	Matrix metalloproteinase 12	MMP12	0.122581769
MOL001689	acacetin	Lymphocyte differentiation antigen CD38	CD38	0.122581769
MOL001689	acacetin	DNA topoisomerase I (by homology)	TOP1	0.122581769
MOL001689	acacetin	Arginase-1 (by homology)	ARG1	0.122581769
MOL001689	acacetin	Lysine-specific demethylase 4D-like	KDM4E	0.122581769
MOL001689	acacetin	Arachidonate 15-lipoxygenase	ALOX15	0.122581769
MOL001689	acacetin	Cyclin-dependent kinase 1	CDK1	0.122581769
MOL001689	acacetin	Arachidonate 12-lipoxygenase	ALOX12	0.122581769
MOL001689	acacetin	Phospholipase A2 group IIA	PLA2G2A	0.106099949
MOL001689	acacetin	NEDD8-activating enzyme E1 regulatory subunit	NAE1	0.106099949
MOL001689	acacetin	6-phosphofructo-2-kinase/fructose-2,6-bisphosphatase 3	PFKFB3	0.106099949
MOL001689	acacetin	Tyrosinase	TYR	0.106099949
MOL001689	acacetin	Estradiol 17-beta-dehydrogenase 2	HSD17B2	0.106099949
MOL001689	acacetin	Aryl hydrocarbon receptor	AHR	0.106099949
MOL001689	acacetin	Estrogen-related receptor alpha	ESRRA	0.106099949
MOL001689	acacetin	Tyrosine-protein kinase LCK	LCK	0.106099949
MOL001689	acacetin	Induced myeloid leukemia cell differentiation protein Mcl-1	MCL1	0.106099949
MOL001689	acacetin	Telomerase reverse transcriptase	TERT	0.106099949
MOL001689	acacetin	Nitric oxide synthase, inducible (by homology)	NOS2	0.106099949
MOL001689	acacetin	Monoamine oxidase B	MAOB	0.097874534
MOL001689	acacetin	Butyrylcholinesterase	BCHE	0.097874534
MOL001689	acacetin	G-protein coupled receptor 35	GPR35	0.097874534
MOL001689	acacetin	Death-associated protein kinase 1	DAPK1	0.097874534
MOL001689	acacetin	DNA-3-methyladenine glycosylase	MPG	0.097874534
MOL001689	acacetin	Solute carrier family 22 member 12	SLC22A12	0.097874534
MOL001689	acacetin	Mu opioid receptor (by homology)	OPRM1	0.097874534
MOL001689	acacetin	Inhibitor of nuclear factor kappa B kinase beta subunit	IKBKB	0.097874534
MOL001689	acacetin	Neurotrophic tyrosine kinase receptor type 2	NTRK2	0.097874534
MOL001689	acacetin	Beta-galactoside alpha-2,6-sialyltransferase 1	ST6GAL1	0.097874534

MOL001689	acacetin	Sigma opioid receptor	SIGMAR1	0.097874534
MOL001689	acacetin	Microtubule-associated protein tau	MAPT	0.097874534
MOL001689	acacetin	Vasopressin V2 receptor	AVPR2	0.097874534
MOL001689	acacetin	DNA topoisomerase II alpha	TOP2A	0.097874534
MOL001689	acacetin	Insulin-like growth factor I receptor	IGF1R	0.097874534
MOL001689	acacetin	Insulin receptor	INSR	0.097874534
MOL001689	acacetin	Epidermal growth factor receptor erbB1	EGFR	0.097874534
MOL001689	acacetin	Thrombin	F2	0.097874534
MOL001689	acacetin	Serine/threonine-protein kinase Aurora-B	AURKB	0.097874534
MOL001689	acacetin	Dopamine D4 receptor	DRD4	0.097874534
MOL001689	acacetin	Myosin light chain kinase, smooth muscle	MYLK	0.097874534
MOL001689	acacetin	Myeloperoxidase	MPO	0.097874534
MOL001689	acacetin	PI3-kinase p85-alpha subunit	PIK3R1	0.097874534
MOL001689	acacetin	Liver glycogen phosphorylase	PYGL	0.097874534
MOL001689	acacetin	Tyrosine-protein kinase SRC	SRC	0.097874534
MOL001689	acacetin	Focal adhesion kinase 1	PTK2	0.097874534
MOL001689	acacetin	Vascular endothelial growth factor receptor 2	KDR	0.097874534
MOL001689	acacetin	Matrix metalloproteinase 13	MMP13	0.097874534
MOL001689	acacetin	Matrix metalloproteinase 3	MMP3	0.097874534
MOL001689	acacetin	Carbonic anhydrase III	CA3	0.097874534
MOL001689	acacetin	Serine/threonine-protein kinase PLK1	PLK1	0.097874534
MOL000173	wogonin	Cyclooxygenase-2 (by homology)	PTGS2	0.320218721
MOL000173	wogonin	Nitric oxide synthase, inducible (by homology)	NOS2	0.320218721
MOL000173	wogonin	Aldose reductase (by homology)	AKR1B1	0.270836923
MOL000173	wogonin	Delta opioid receptor (by homology)	OPRD1	0.147256737
MOL000173	wogonin	Mu opioid receptor (by homology)	OPRM1	0.147256737
MOL000173	wogonin	Stem cell growth factor receptor	KIT	0.139061947
MOL000173	wogonin	Tyrosine-protein kinase receptor FLT3	FLT3	0.114337559
MOL000173	wogonin	6-phosphofructo-2-kinase/fructose-2,6-bisphosphatase 3	PFKFB3	0.114337559
MOL000173	wogonin	Receptor-type tyrosine-protein phosphatase S	PTPRS	0.114337559
MOL000173	wogonin	Serine/threonine-protein kinase PIM1	PIM1	0.106099949
MOL000173	wogonin	Adenosine A1 receptor (by homology)	ADORA1	0.106099949
MOL000173	wogonin	Adenosine A2a receptor (by homology)	ADORA2A	0.106099949
MOL000173	wogonin	P-glycoprotein 1	ABCB1	0.106099949
MOL000173	wogonin	ATP-binding cassette sub-family G member 2	ABCG2	0.106099949
MOL000173	wogonin	Inhibitor of nuclear factor kappa B kinase beta subunit	IKBKB	0.106099949
MOL000173	wogonin	Neurotrophic tyrosine kinase receptor type 2	NTRK2	0.106099949
MOL000173	wogonin	Cytochrome P450 1A1	CYP1A1	0.106099949
MOL000173	wogonin	Cytochrome P450 1A2	CYP1A2	0.106099949
MOL000173	wogonin	Cytochrome P450 1B1	CYP1B1	0.106099949
MOL000173	wogonin	Telomerase reverse transcriptase	TERT	0.106099949
MOL000173	wogonin	Induced myeloid leukemia cell differentiation protein	MCL1	0.106099949

		Mcl-1		
MOL000173	wogonin	Arachidonate 5-lipoxygenase	ALOX5	0.106099949
MOL000173	wogonin	Adenosine A3 receptor	ADORA3	0.106099949
MOL000173	wogonin	Lysine-specific demethylase 4D-like	KDM4E	0.097874534
MOL000173	wogonin	Xanthine dehydrogenase	XDH	0.097874534
MOL000173	wogonin	Arachidonate 15-lipoxygenase	ALOX15	0.097874534
MOL000173	wogonin	Cyclin-dependent kinase 1	CDK1	0.097874534
MOL000173	wogonin	Arachidonate 12-lipoxygenase	ALOX12	0.097874534
MOL000173	wogonin	G protein-coupled receptor kinase 6	GRK6	0.097874534
MOL000173	wogonin	Butyrylcholinesterase	BCHE	0.097874534
MOL000173	wogonin	Acetylcholinesterase	ACHE	0.097874534
MOL000173	wogonin	Carbonic anhydrase VII	CA7	0.097874534
MOL000173	wogonin	Carbonic anhydrase XII	CA12	0.097874534
MOL000173	wogonin	Carbonic anhydrase IV	CA4	0.097874534
MOL000173	wogonin	Cyclin-dependent kinase 5/CDK5 activator 1	CDK5R1	0.097874534
MOL000173	wogonin	Cytochrome P450 19A1	CYP19A1	0.097874534
MOL000173	wogonin	Carbonic anhydrase II	CA2	0.097874534
MOL000173	wogonin	Cyclin-dependent kinase 1/cyclin B	CCNB3	0.097874534
MOL000173	wogonin	Cyclin-dependent kinase 6	CDK6	0.097874534
MOL000173	wogonin	Carbonic anhydrase I	CA1	0.097874534
MOL000173	wogonin	Carbonic anhydrase IX	CA9	0.097874534
MOL000173	wogonin	Carbonyl reductase [NADPH] 1	CBR1	0.097874534
MOL000173	wogonin	Multidrug resistance-associated protein 1	ABCC1	0.097874534
MOL000173	wogonin	Tankyrase-1	TNKS	0.097874534
MOL000173	wogonin	AMY1C	AMY1A	0.097874534
MOL000173	wogonin	Beta amyloid A4 protein	APP	0.097874534
MOL000173	wogonin	NADPH oxidase 4	NOX4	0.097874534
MOL000173	wogonin	Tyrosinase	TYR	0.097874534
MOL000173	wogonin	Estradiol 17-beta-dehydrogenase 2	HSD17B2	0.097874534
MOL000173	wogonin	Estradiol 17-beta-dehydrogenase 1	HSD17B1	0.097874534
MOL000173	wogonin	Aryl hydrocarbon receptor	AHR	0.097874534
MOL000173	wogonin	Estrogen-related receptor alpha	ESRRA	0.097874534
MOL000173	wogonin	Tankyrase-2	TNKS2	0.097874534
MOL000173	wogonin	Monoamine oxidase A	MAOA	0.097874534
MOL000173	wogonin	Estrogen receptor alpha	ESR1	0.097874534
MOL000173	wogonin	Estrogen receptor beta	ESR2	0.097874534
MOL000173	wogonin	Tyrosine-protein kinase SYK	SYK	0.097874534
MOL000173	wogonin	Glycogen synthase kinase-3 beta	GSK3B	0.097874534
MOL000173	wogonin	Transthyretin	TTR	0.097874534
MOL000173	wogonin	Casein kinase II alpha	CSNK2A1	0.097874534
MOL000173	wogonin	Cystic fibrosis transmembrane conductance regulator	CFTR	0.097874534
MOL000173	wogonin	Aldo-keto reductase family 1 member B10	AKR1B10	0.097874534
MOL000173	wogonin	Plasminogen	PLG	0.097874534

MOL000173	wogonin	Androgen Receptor	AR	0.097874534
MOL000173	wogonin	G-protein coupled receptor 35	GPR35	0.097874534
MOL000173	wogonin	Death-associated protein kinase 1	DAPK1	0.097874534
MOL000173	wogonin	DNA-3-methyladenine glycosylase	MPG	0.097874534
MOL000173	wogonin	Solute carrier family 22 member 12	SLC22A12	0.097874534
MOL000173	wogonin	Glyoxalase I	GLO1	0.097874534
MOL000173	wogonin	Poly [ADP-ribose] polymerase-1	PARP1	0.097874534
MOL000173	wogonin	Matrix metalloproteinase 9	MMP9	0.097874534
MOL000173	wogonin	Matrix metalloproteinase 2	MMP2	0.097874534
MOL000173	wogonin	Matrix metalloproteinase 12	MMP12	0.097874534
MOL000173	wogonin	Lymphocyte differentiation antigen CD38	CD38	0.097874534
MOL000173	wogonin	DNA topoisomerase I (by homology)	TOP1	0.097874534
MOL000173	wogonin	Arginase-1 (by homology)	ARG1	0.097874534
MOL000173	wogonin	Microtubule-associated protein tau	MAPT	0.097874534
MOL000173	wogonin	Vasopressin V2 receptor	AVPR2	0.097874534
MOL000173	wogonin	DNA topoisomerase II alpha	TOP2A	0.097874534
MOL000173	wogonin	Insulin-like growth factor I receptor	IGF1R	0.097874534
MOL000173	wogonin	Insulin receptor	INSR	0.097874534
MOL000173	wogonin	Epidermal growth factor receptor erbB1	EGFR	0.097874534
MOL000173	wogonin	Thrombin	F2	0.097874534
MOL000173	wogonin	Serine/threonine-protein kinase Aurora-B	AURKB	0.097874534
MOL000173	wogonin	Dopamine D4 receptor	DRD4	0.097874534
MOL000173	wogonin	Myosin light chain kinase, smooth muscle	MYLK	0.097874534
MOL000173	wogonin	Myeloperoxidase	MPO	0.097874534
MOL000173	wogonin	PI3-kinase p85-alpha subunit	PIK3R1	0.097874534
MOL000173	wogonin	Liver glycogen phosphorylase	PYGL	0.097874534
MOL000173	wogonin	Tyrosine-protein kinase SRC	SRC	0.097874534
MOL000173	wogonin	Focal adhesion kinase 1	PTK2	0.097874534
MOL000173	wogonin	Vascular endothelial growth factor receptor 2	KDR	0.097874534
MOL000173	wogonin	Matrix metalloproteinase 13	MMP13	0.097874534
MOL000173	wogonin	Matrix metalloproteinase 3	MMP3	0.097874534
MOL000173	wogonin	Carbonic anhydrase III	CA3	0.097874534
MOL000173	wogonin	Serine/threonine-protein kinase PLK1	PLK1	0.097874534
MOL000173	wogonin	Carbonic anhydrase VI	CA6	0.097874534
MOL000173	wogonin	PI3-kinase p110-gamma subunit	PIK3CG	0.097874534
MOL000173	wogonin	Protein kinase N1	PKN1	0.097874534
MOL000173	wogonin	Carbonic anhydrase XIV	CA14	0.097874534
MOL000228	Alpinetin	ATP-binding cassette sub-family G member 2	ABCG2	0.28080856
MOL000228	Alpinetin	Cytochrome P450 19A1	CYP19A1	0.28080856
MOL000228	Alpinetin	Cytochrome P450 1B1	CYP1B1	0.28080856
MOL000228	Alpinetin	Taste receptor type 2 member 31	TAS2R31	0.25622784
MOL000228	Alpinetin	Adenosine A3 receptor	ADORA3	0.25622784
MOL000228	Alpinetin	Adenosine A1 receptor (by homology)	ADORA1	0.25622784
