# Supplementary material for: Exploring the Mechanism of Scutellaria baicalensis Georgi Efficacy against Oral Squamous Cell Carcinoma Based on Network Pharmacology and Molecular Docking Analysis
Source: Evid Based Complement Alternat Med. 2021 Jul 13;2021:5597586. doi: 10.1155/2021/5597586 (PMC8292061; doi:10.1155/2021/5597586)
Supplement: Supplementary Materials — Table S1: detailed information of active compounds in SBG. Table S2: target gene-related active compounds of SBG. Table S3: list of OSCC-related genes in the GeneCards database, OMIM, and TTD. Table S4: the putative targets of SBG against OSCC. Table S5: topological analysis of the PPI network. Table S6: topological analysis of the compound-target-disease network. Table S7: the GO enrichment analysis for intersection targets between compound and OSCC-related targets. Table S8: the enriched KEGG pathways for intersection targets between compound and AD-related targets. Table S9: the results of molecular docking. [file 5597586.f1.zip › 5597586.f1/Supplementary File 3. OSCC-related genes screened from GeneCards,OMIM and TTD databases.pdf]

Table S3. OSCC-related genes screened from GeneCards,OMIM and TTD databases.

Gene Symbol	Description	Source
TP53	Tumor Protein P53	GeneCards
EGFR	Epidermal Growth Factor Receptor	GeneCards
CDKN2A	Cyclin Dependent Kinase Inhibitor 2A	GeneCards
PTEN	Phosphatase And Tensin Homolog	GeneCards
PIK3CA	Phosphatidylinositol-4,5-Bisphosphate 3-Kinase Catalytic Subunit Alpha	GeneCards
HRAS	HRas Proto-Oncogene, GTPase	GeneCards
CTNNB1	Catenin Beta 1	GeneCards
CCND1	Cyclin D1	GeneCards
CDH1	Cadherin 1	GeneCards
AKT1	AKT Serine/Threonine Kinase 1	GeneCards
MET	MET Proto-Oncogene, Receptor Tyrosine Kinase	GeneCards
ERBB2	Erb-B2 Receptor Tyrosine Kinase 2	GeneCards
MYC	MYC Proto-Oncogene, BHLH Transcription Factor	GeneCards
IL6	Interleukin 6	GeneCards
KRAS	KRAS Proto-Oncogene, GTPase	GeneCards
BRAF	B-Raf Proto-Oncogene, Serine/Threonine Kinase	GeneCards
TNF	Tumor Necrosis Factor	GeneCards
STAT3	Signal Transducer And Activator Of Transcription 3	GeneCards
TP63	Tumor Protein P63	GeneCards
KIT	KIT Proto-Oncogene, Receptor Tyrosine Kinase	GeneCards
TERT	Telomerase Reverse Transcriptase	GeneCards
CDKN1B	Cyclin Dependent Kinase Inhibitor 1B	GeneCards
FAS	Fas Cell Surface Death Receptor	GeneCards
FGFR3	Fibroblast Growth Factor Receptor 3	GeneCards
TGFB1	Transforming Growth Factor Beta 1	GeneCards
CDKN1A	Cyclin Dependent Kinase Inhibitor 1A	GeneCards
APC	APC Regulator Of WNT Signaling Pathway	GeneCards
TGFBR2	Transforming Growth Factor Beta Receptor 2	GeneCards
EGF	Epidermal Growth Factor	GeneCards
RB1	RB Transcriptional Corepressor 1	GeneCards
BCL2	BCL2 Apoptosis Regulator	GeneCards
MTOR	Mechanistic Target Of Rapamycin Kinase	GeneCards
SMAD4	SMAD Family Member 4	GeneCards
IFNG	Interferon Gamma	GeneCards
KRT5	Keratin 5	GeneCards
FASLG	Fas Ligand	GeneCards
KRT14	Keratin 14	GeneCards
IL10	Interleukin 10	GeneCards
CASP8	Caspase 8	GeneCards

BAX	BCL2 Associated X, Apoptosis Regulator	GeneCards
FGFR2	Fibroblast Growth Factor Receptor 2	GeneCards
MIR21	MicroRNA 21	GeneCards
MLH1	MutL Homolog 1	GeneCards
MMP1	Matrix Metallopeptidase 1	GeneCards
BRCA2	BRCA2 DNA Repair Associated	GeneCards
STAT1	Signal Transducer And Activator Of Transcription 1	GeneCards
STK11	Serine/Threonine Kinase 11	GeneCards
CXCR4	C-X-C Motif Chemokine Receptor 4	GeneCards
ATM	ATM Serine/Threonine Kinase	GeneCards
KDR	Kinase Insert Domain Receptor	GeneCards
IL1B	Interleukin 1 Beta	GeneCards
FHIT	Fragile Histidine Triad Diadenosine Triphosphatase	GeneCards
CDK4	Cyclin Dependent Kinase 4	GeneCards
RET	Ret Proto-Oncogene	GeneCards
PTCH1	Patched 1	GeneCards
MDM2	MDM2 Proto-Oncogene	GeneCards
H19	H19 Imprinted Maternally Expressed Transcript	GeneCards
NFE2L2	Nuclear Factor, Erythroid 2 Like 2	GeneCards
TGFA	Transforming Growth Factor Alpha	GeneCards
SRC	SRC Proto-Oncogene, Non-Receptor Tyrosine Kinase	GeneCards
BRCA1	BRCA1 DNA Repair Associated	GeneCards
NRAS	NRAS Proto-Oncogene, GTPase	GeneCards
MSH2	MutS Homolog 2	GeneCards
ESR1	Estrogen Receptor 1	GeneCards
CTLA4	Cytotoxic T-Lymphocyte Associated Protein 4	GeneCards
CD4	CD4 Molecule	GeneCards
VEGFA	Vascular Endothelial Growth Factor A	GeneCards
FGFR1	Fibroblast Growth Factor Receptor 1	GeneCards
EPCAM	Epithelial Cell Adhesion Molecule	GeneCards
CHEK2	Checkpoint Kinase 2	GeneCards
PTPRC	Protein Tyrosine Phosphatase Receptor Type C	GeneCards
CDKN2B	Cyclin Dependent Kinase Inhibitor 2B	GeneCards
SETD2	SET Domain Containing 2, Histone Lysine Methyltransferase	GeneCards
VEGFC	Vascular Endothelial Growth Factor C	GeneCards
MIR31	MicroRNA 31	GeneCards
CD8A	CD8a Molecule	GeneCards
TNFRSF10B	TNF Receptor Superfamily Member 10b	GeneCards
CXCL12	C-X-C Motif Chemokine Ligand 12	GeneCards
SLC2A1	Solute Carrier Family 2 Member 1	GeneCards
MIR145	MicroRNA 145	GeneCards
B2M	Beta-2-Microglobulin	GeneCards
U2AF1	U2 Small Nuclear RNA Auxiliary Factor 1	GeneCards

FLT1	Fms Related Receptor Tyrosine Kinase 1	GeneCards
MAP2K1	Mitogen-Activated Protein Kinase Kinase 1	GeneCards
ERBB3	Erb-B2 Receptor Tyrosine Kinase 3	GeneCards
JAK3	Janus Kinase 3	GeneCards
SPP1	Secreted Phosphoprotein 1	GeneCards
MIR155	MicroRNA 155	GeneCards
MIR17	MicroRNA 17	GeneCards
PDCD1	Programmed Cell Death 1	GeneCards
FN1	Fibronectin 1	GeneCards
INS	Insulin	GeneCards
PPARG	Peroxisome Proliferator Activated Receptor Gamma	GeneCards
VHL	Von Hippel-Lindau Tumor Suppressor	GeneCards
DNMT3B	DNA Methyltransferase 3 Beta	GeneCards
TERC	Telomerase RNA Component	GeneCards
TYMP	Thymidine Phosphorylase	GeneCards
OGG1	8-Oxoguanine DNA Glycosylase	GeneCards
NKX2-1	NK2 Homeobox 1	GeneCards
MIR143	MicroRNA 143	GeneCards
EP300	E1A Binding Protein P300	GeneCards
IGF2	Insulin Like Growth Factor 2	GeneCards
FLT4	Fms Related Receptor Tyrosine Kinase 4	GeneCards
ICAM1	Intercellular Adhesion Molecule 1	GeneCards
TP73	Tumor Protein P73	GeneCards
CDH2	Cadherin 2	GeneCards
MMP9	Matrix Metallopeptidase 9	GeneCards
CASP3	Caspase 3	GeneCards
PTGS2	Prostaglandin-Endoperoxide Synthase 2	GeneCards
MMP2	Matrix Metallopeptidase 2	GeneCards
MIR221	MicroRNA 221	GeneCards
CD44	CD44 Molecule (Indian Blood Group)	GeneCards
ALK	ALK Receptor Tyrosine Kinase	GeneCards
WT1	WT1 Transcription Factor	GeneCards
ERCC2	ERCC Excision Repair 2, TFIIH Core Complex Helicase Subunit	GeneCards
DNMT1	DNA Methyltransferase 1	GeneCards
SHH	Sonic Hedgehog Signaling Molecule	GeneCards
CCL2	C-C Motif Chemokine Ligand 2	GeneCards
PDGFRA	Platelet Derived Growth Factor Receptor Alpha	GeneCards
RAF1	Raf-1 Proto-Oncogene, Serine/Threonine Kinase	GeneCards
HLA-DRB1	Major Histocompatibility Complex, Class II, DR Beta 1	GeneCards
ENG	Endoglin	GeneCards
NFKBIA	NFKB Inhibitor Alpha	GeneCards
MGMT	O-6-Methylguanine-DNA Methyltransferase	GeneCards
MIR27A	MicroRNA 27a	GeneCards

IL7	Interleukin 7	GeneCards
PDGFRB	Platelet Derived Growth Factor Receptor Beta	GeneCards
KRT13	Keratin 13	GeneCards
MIR34A	MicroRNA 34a	GeneCards
PIK3R1	Phosphoinositide-3-Kinase Regulatory Subunit 1	GeneCards
MIR142	MicroRNA 142	GeneCards
ING1	Inhibitor Of Growth Family Member 1	GeneCards
TGFBR1	Transforming Growth Factor Beta Receptor 1	GeneCards
MIR195	MicroRNA 195	GeneCards
TLR4	Toll Like Receptor 4	GeneCards
HSPB1	Heat Shock Protein Family B (Small) Member 1	GeneCards
MIR205	MicroRNA 205	GeneCards
XIAP	X-Linked Inhibitor Of Apoptosis	GeneCards
HIF1A	Hypoxia Inducible Factor 1 Subunit Alpha	GeneCards
FGFR4	Fibroblast Growth Factor Receptor 4	GeneCards
ERCC6	ERCC Excision Repair 6, Chromatin Remodeling Factor	GeneCards
GNAS	GNAS Complex Locus	GeneCards
NBN	Nibrin	GeneCards
CXCL8	C-X-C Motif Chemokine Ligand 8	GeneCards
MAPK1	Mitogen-Activated Protein Kinase 1	GeneCards
LAMC2	Laminin Subunit Gamma 2	GeneCards
MIR140	MicroRNA 140	GeneCards
JAK2	Janus Kinase 2	GeneCards
MUC1	Mucin 1, Cell Surface Associated	GeneCards
CAV1	Caveolin 1	GeneCards
SMAD3	SMAD Family Member 3	GeneCards
PTPN11	Protein Tyrosine Phosphatase Non-Receptor Type 11	GeneCards
ABCG2	ATP Binding Cassette Subfamily G Member 2 (Junior Blood Group)	GeneCards
IL2	Interleukin 2	GeneCards
PLAU	Plasminogen Activator, Urokinase	GeneCards
ITGA6	Integrin Subunit Alpha 6	GeneCards
NOTCH1	Notch Receptor 1	GeneCards
MIR144	MicroRNA 144	GeneCards
RELA	RELA Proto-Oncogene, NF-KB Subunit	GeneCards
ABL1	ABL Proto-Oncogene 1, Non-Receptor Tyrosine Kinase	GeneCards
MIR126	MicroRNA 126	GeneCards
LOX	Lysyl Oxidase	GeneCards
AR	Androgen Receptor	GeneCards
KRT19	Keratin 19	GeneCards
SMARCA4	SWI/SNF Related, Matrix Associated, Actin Dependent Regulator Of Chromatin, Subfamily A, Member 4	GeneCards
FOS	Fos Proto-Oncogene, AP-1 Transcription Factor Subunit	GeneCards
NTRK1	Neurotrophic Receptor Tyrosine Kinase 1	GeneCards

HLA-A	Major Histocompatibility Complex, Class I, A	GeneCards
SNAI2	Snail Family Transcriptional Repressor 2	GeneCards
ERCC1	ERCC Excision Repair 1, Endonuclease Non-Catalytic Subunit	GeneCards
MIR141	MicroRNA 141	GeneCards
MIR204	MicroRNA 204	GeneCards
FOXP3	Forkhead Box P3	GeneCards
MIR200A	MicroRNA 200a	GeneCards
NFKB1	Nuclear Factor Kappa B Subunit 1	GeneCards
MIR193A	MicroRNA 193a	GeneCards
FGF2	Fibroblast Growth Factor 2	GeneCards
MIR150	MicroRNA 150	GeneCards
IDH2	Isocitrate Dehydrogenase (NADP(+)) 2	GeneCards
CD274	CD274 Molecule	GeneCards
MIR181A1	MicroRNA 181a-1	GeneCards
MIR125A	MicroRNA 125a	GeneCards
PROM1	Prominin 1	GeneCards
MIR483	MicroRNA 483	GeneCards
MIR214	MicroRNA 214	GeneCards
MIR146A	MicroRNA 146a	GeneCards
BMP4	Bone Morphogenetic Protein 4	GeneCards
RAD51	RAD51 Recombinase	GeneCards
WWOX	WW Domain Containing Oxidoreductase	GeneCards
MIR15A	MicroRNA 15a	GeneCards
MIR200B	MicroRNA 200b	GeneCards
MSH6	MutS Homolog 6	GeneCards
MIR99A	MicroRNA 99a	GeneCards
MAP2K2	Mitogen-Activated Protein Kinase Kinase 2	GeneCards
MIR30E	MicroRNA 30e	GeneCards
MIR22	MicroRNA 22	GeneCards
RARA	Retinoic Acid Receptor Alpha	GeneCards
ITGB4	Integrin Subunit Beta 4	GeneCards
MIR210	MicroRNA 210	GeneCards
HLA-B	Major Histocompatibility Complex, Class I, B	GeneCards
MIR486-1	MicroRNA 486-1	GeneCards
BMP2	Bone Morphogenetic Protein 2	GeneCards
ACTB	Actin Beta	GeneCards
BIRC5	Baculoviral IAP Repeat Containing 5	GeneCards
KRT7	Keratin 7	GeneCards
MIR148A	MicroRNA 148a	GeneCards
MIR200C	MicroRNA 200c	GeneCards
MIR185	MicroRNA 185	GeneCards
GATA2	GATA Binding Protein 2	GeneCards
JUN	Jun Proto-Oncogene, AP-1 Transcription Factor Subunit	GeneCards

MIR29C	MicroRNA 29c	GeneCards
BIRC3	Baculoviral IAP Repeat Containing 3	GeneCards
KRT17	Keratin 17	GeneCards
TYR	Tyrosinase	GeneCards
CYP1A1	Cytochrome P450 Family 1 Subfamily A Member 1	GeneCards
MIR223	MicroRNA 223	GeneCards
GLI1	GLI Family Zinc Finger 1	GeneCards
HNF1B	HNF1 Homeobox B	GeneCards
IKBKB	Inhibitor Of Nuclear Factor Kappa B Kinase Subunit Beta	GeneCards
MIR424	MicroRNA 424	GeneCards
XPA	XPA, DNA Damage Recognition And Repair Factor	GeneCards
MIR18A	MicroRNA 18a	GeneCards
SMARCB1	SWI/SNF Related, Matrix Associated, Actin Dependent Regulator Of Chromatin, Subfamily B, Member 1	GeneCards
DPYD	Dihydropyrimidine Dehydrogenase	GeneCards
ESR2	Estrogen Receptor 2	GeneCards
LAMB3	Laminin Subunit Beta 3	GeneCards
BCL2L1	BCL2 Like 1	GeneCards
BCL10	BCL10 Immune Signaling Adaptor	GeneCards
KRT10	Keratin 10	GeneCards
IL4	Interleukin 4	GeneCards
MIR19A	MicroRNA 19a	GeneCards
PSMB8	Proteasome 20S Subunit Beta 8	GeneCards
CAT	Catalase	GeneCards
NOS2	Nitric Oxide Synthase 2	GeneCards
FANCC	FA Complementation Group C	GeneCards
DDB2	Damage Specific DNA Binding Protein 2	GeneCards
GSTP1	Glutathione S-Transferase Pi 1	GeneCards
AKT2	AKT Serine/Threonine Kinase 2	GeneCards
CD40LG	CD40 Ligand	GeneCards
JUP	Junction Plakoglobin	GeneCards
MIR29A	MicroRNA 29a	GeneCards
GSTM1	Glutathione S-Transferase Mu 1	GeneCards
IGF1	Insulin Like Growth Factor 1	GeneCards
TLR2	Toll Like Receptor 2	GeneCards
ERBB4	Erb-B2 Receptor Tyrosine Kinase 4	GeneCards
MKI67	Marker Of Proliferation Ki-67	GeneCards
EPOR	Erythropoietin Receptor	GeneCards
IL2RA	Interleukin 2 Receptor Subunit Alpha	GeneCards
DKC1	Dyskerin Pseudouridine Synthase 1	GeneCards
CDK6	Cyclin Dependent Kinase 6	GeneCards
CCR6	C-C Motif Chemokine Receptor 6	GeneCards
CDKN3	Cyclin Dependent Kinase Inhibitor 3	GeneCards
