# Supplementary material for: Exploring the Mechanism of Scutellaria baicalensis Georgi Efficacy against Oral Squamous Cell Carcinoma Based on Network Pharmacology and Molecular Docking Analysis
Source: Evid Based Complement Alternat Med. 2021 Jul 13;2021:5597586. doi: 10.1155/2021/5597586 (PMC8292061; doi:10.1155/2021/5597586)
Supplement: Supplementary Materials — Table S1: detailed information of active compounds in SBG. Table S2: target gene-related active compounds of SBG. Table S3: list of OSCC-related genes in the GeneCards database, OMIM, and TTD. Table S4: the putative targets of SBG against OSCC. Table S5: topological analysis of the PPI network. Table S6: topological analysis of the compound-target-disease network. Table S7: the GO enrichment analysis for intersection targets between compound and OSCC-related targets. Table S8: the enriched KEGG pathways for intersection targets between compound and AD-related targets. Table S9: the results of molecular docking. [file 5597586.f1.zip › 5597586.f1/Supplementary File 4. The putative targets of SBG against OSCC.pdf]

Table S4. The putative targets of SBG against OSCC.

Gene Symbol	Description
MMP2	Matrix metalloproteinase 2
ABCB1	P-glycoprotein 1
ABCC1	Multidrug resistance-associated protein 1
ABCG2	ATP-binding cassette sub-family G member 2
ACHE	Acetylcholinesterase (Cartwright Blood Group)
AHR	Aryl hydrocarbon receptor
AKT1	AKT Serine/Threonine Kinase 1
ALK	ALK Receptor Tyrosine Kinase
ALOX12	Arachidonate 12-lipoxygenase
ALOX5	Arachidonate 5-lipoxygenase
APEX1	Apurinic/Apyrimidinic Endodeoxyribonuclease 1
APP	Amyloid Beta Precursor Protein
AR	Androgen Receptor
AURKB	Serine/threonine-protein kinase Aurora-B
AXL	AXL Receptor Tyrosine Kinase
BCL2	Apoptosis regulator Bcl-2
CA2	Carbonic anhydrase II
CA9	Carbonic anhydrase IX
CDC42	Cell division control protein 42 homolog
CDK1	Cyclin-dependent kinase 1
CDK2	Cyclin-dependent kinase 2
CDK6	Cyclin-dependent kinase 6
CFTR	Cystic fibrosis transmembrane conductance regulator
CTSB	Cathepsin B
CXCR1	C-X-C Motif Chemokine Receptor 1
CYP19A1	Cytochrome P450 Family 19 Subfamily A Member 1
CYP1A1	Cytochrome P450 Family 1 Subfamily A Member 1
CYP1B1	Cytochrome P450 Family 1 Subfamily B Member 1
CYP2D6	Cytochrome P450 Family 2 Subfamily D Member 6
DAPK1	Death Associated Protein Kinase 1
DNMT1	DNA Methyltransferase 1
DRD2	Dopamine Receptor D2
EDNRA	Endothelin Receptor Type A
EGFR	Epidermal growth factor receptor
ESR2	Estrogen Receptor 2
F2	Coagulation Factor II, Thrombin
FGFR1	Fibroblast Growth Factor Receptor 1
FLT3	Fms Related Receptor Tyrosine Kinase 3
GSK3B	Glycogen Synthase Kinase 3 Beta
HIF1A	Hypoxia Inducible Factor 1 Subunit Alpha

HNF4A	Hepatocyte Nuclear Factor 4 Alpha
IGF1R	Insulin Like Growth Factor 1 Receptor
IKBKB	Inhibitor Of Nuclear Factor Kappa B Kinase Subunit Beta
INSR	Insulin Receptor
KDR	Kinase Insert Domain Receptor
KIT	KIT Ligand
LCK	LCK Proto-Oncogene, Src Family Tyrosine Kinase
MAPK14	Mitogen-Activated Protein Kinase 14
MAPK3	Mitogen-Activated Protein Kinase 3
MCL1	MCL1 Apoptosis Regulator, BCL2 Family Member
MET	Hepatocyte growth factor receptor
MMP13	Matrix Metallopeptidase 13
MMP14	Matrix Metallopeptidase 14
MMP3	Matrix Metallopeptidase 3
MMP9	Matrix Metallopeptidase 9
MPO	Myeloperoxidase
MYLK	Myosin Light Chain Kinase
NOS2	Nitric Oxide Synthase 2
NR1H4	Nuclear Receptor Subfamily 1 Group H Member 4
NTRK2	Neurotrophic Receptor Tyrosine Kinase 2
ODC1	Ornithine Decarboxylase 1
PARP1	Poly(ADP-Ribose) Polymerase 1
PGF	Placental Growth Factor
PIK3CG	Phosphatidylinositol-4,5-Bisphosphate 3-Kinase Catalytic Subunit Gamma
PIK3R1	Phosphoinositide-3-Kinase Regulatory Subunit 1
PLG	Plasminogen
PLK1	Polo Like Kinase 1
PPARG	PPARG Coactivator 1 Alpha
PTGS1	Prostaglandin-Endoperoxide Synthase 1
PTGS2	Prostaglandin-Endoperoxide Synthase 2
PTK2	Protein Tyrosine Kinase 2
PTPN1	Protein Tyrosine Phosphatase Non-Receptor Type 1
RAC1	Rac Family Small GTPase 1
RXRA	Retinoid X Receptor Alpha
SERPINE1	Serpin Family E Member 1
SHBG	Sex Hormone Binding Globulin
SRC	SRC Proto-Oncogene, Non-Receptor Tyrosine Kinase
STAT1	Signal Transducer And Activator Of Transcription 1
SYK	Spleen Associated Tyrosine Kinase
TERT	Telomerase Reverse Transcriptase
TNF	Tumor Necrosis Factor
TOP1	DNA Topoisomerase I
TOP2A	DNA Topoisomerase II Alpha

TTR	Transthyretin
TYR	Tyrosinase
VEGFA	Vascular Endothelial Growth Factor A
