# Supplementary material for: Exploring the Mechanism of Scutellaria baicalensis Georgi Efficacy against Oral Squamous Cell Carcinoma Based on Network Pharmacology and Molecular Docking Analysis
Source: Evid Based Complement Alternat Med. 2021 Jul 13;2021:5597586. doi: 10.1155/2021/5597586 (PMC8292061; doi:10.1155/2021/5597586)
Supplement: Supplementary Materials — Table S1: detailed information of active compounds in SBG. Table S2: target gene-related active compounds of SBG. Table S3: list of OSCC-related genes in the GeneCards database, OMIM, and TTD. Table S4: the putative targets of SBG against OSCC. Table S5: topological analysis of the PPI network. Table S6: topological analysis of the compound-target-disease network. Table S7: the GO enrichment analysis for intersection targets between compound and OSCC-related targets. Table S8: the enriched KEGG pathways for intersection targets between compound and AD-related targets. Table S9: the results of molecular docking. [file 5597586.f1.zip › 5597586.f1/Supplementary File 5. Topological analysis of the PPI network.pdf]

Table S5. Topological analysis of the PPI network.

Gene name	AverageShortestPathLength	BetweennessCentrality	ClosenessCentrality	Degree
PIK3R1	1.95774648	0.15354303	0.51079137	29
SRC	1.97183099	0.12269507	0.50714286	25
AKT1	1.95774648	0.17433512	0.51079137	25
MAPK3	2.04225352	0.15869705	0.48965517	21
VEGFA	1.98591549	0.16270005	0.5035461	19
CDC42	2.14084507	0.0674869	0.46710526	17
FYN	2.15492958	0.01987382	0.46405229	17
RAC1	2.23943662	0.01217278	0.44654088	16
EGFR	2.16901408	0.04929797	0.46103896	15
LCK	2.26760563	0.00724493	0.44099379	14
MAPK14	2.25352113	0.01197521	0.44375	13
PTK2	2.23943662	0.00398923	0.44654088	12
TNF	2.32394366	0.0574115	0.43030303	10
SYK	2.54929577	0.00228265	0.39226519	10
APP	2.28169014	0.12342172	0.4382716	10
IL2	2.38028169	0.00905315	0.42011834	10
STAT1	2.43661972	0.00808071	0.41040462	9
KDR	2.46478873	0.00624244	0.40571429	9
MET	2.33802817	0.00232831	0.42771084	8
RXRA	2.25352113	0.16315743	0.44375	8
MMP9	2.46478873	0.02167976	0.40571429	7
CDK1	2.53521127	0.05897076	0.39444444	7
PTPN1	2.43661972	0.00228874	0.41040462	7
EDNRA	2.38028169	0.00697946	0.42011834	7
MMP2	2.61971831	0.03313263	0.38172043	6
IGF1R	2.3943662	0.00356033	0.41764706	6
KIT	2.5915493	0.00121306	0.38586957	6
ESR2	2.53521127	3.59E-04	0.39444444	6
F2	2.3943662	0.01109357	0.41764706	6
PLG	2.73239437	0.01105709	0.36597938	6
AR	2.43661972	0.0127247	0.41040462	6
CYP1A1	3.12676056	0.10985915	0.31981982	6
MMP13	3.25352113	0.00309469	0.30735931	5
MMP3	2.88732394	0.00449245	0.34634146	5
PTGS2	2.69014085	0.0825747	0.37172775	5
HIF1A	2.43661972	0.02884542	0.41040462	5
PIK3CG	2.6056338	3.37E-04	0.38378378	5
NOS2	2.42253521	0.01985924	0.4127907	5
GSK3B	2.69014085	0.00542867	0.37172775	5

NTRK2	2.70422535	0	0.36979167	4
TOP2A	3.43661972	0.02816901	0.29098361	4
PLK1	2.87323944	0.00960728	0.34803922	4
AURKB	2.87323944	0.00960728	0.34803922	4
CDK6	3.08450704	0.00325416	0.32420091	4
BCL2	2.52112676	0.02816901	0.39664804	4
INSR	2.61971831	2.25E-04	0.38172043	4
CDK2	2.83098592	0.00554796	0.35323383	3
SERPINE1	2.78873239	0	0.35858586	3
ALOX5	3.63380282	0.0138833	0.2751938	3
ALOX12	3.63380282	0.0138833	0.2751938	3
TERT	2.66197183	0	0.37566138	3
PPARG	2.95774648	0	0.33809524	2
MMP14	3.57746479	0	0.27952756	2
CYP19A1	4.11267606	0	0.24315068	2
CFTR	3.05633803	1.31E-04	0.32718894	2
AXL	2.91549296	0	0.34299517	2
DRD2	3.25352113	0	0.30735931	2
CXCR1	3.25352113	0	0.30735931	2
TTR	3.23943662	0.02816901	0.30869565	2
PTGS1	4.6056338	0	0.21712538	2
IKBKB	2.78873239	2.61E-04	0.35858586	2
CYP1B1	4.09859155	0	0.24398625	2
AHR	4.09859155	0	0.24398625	2
TOP1	4.42253521	0	0.22611465	1
MPO	4.22535211	0	0.23666667	1
FLT3	2.94366197	0	0.33971292	1
FGFR1	2.94366197	0	0.33971292	1
DAPK1	3.02816901	0	0.33023256	1
CYP2D6	4.11267606	0	0.24315068	1
CA9	3.42253521	0	0.29218107	1
MCL1	3.50704225	0	0.28514056	1
PRKDC	2.94366197	0	0.33971292	1
