# Supplementary material for: Exploring the Mechanism of Scutellaria baicalensis Georgi Efficacy against Oral Squamous Cell Carcinoma Based on Network Pharmacology and Molecular Docking Analysis
Source: Evid Based Complement Alternat Med. 2021 Jul 13;2021:5597586. doi: 10.1155/2021/5597586 (PMC8292061; doi:10.1155/2021/5597586)
Supplement: Supplementary Materials — Table S1: detailed information of active compounds in SBG. Table S2: target gene-related active compounds of SBG. Table S3: list of OSCC-related genes in the GeneCards database, OMIM, and TTD. Table S4: the putative targets of SBG against OSCC. Table S5: topological analysis of the PPI network. Table S6: topological analysis of the compound-target-disease network. Table S7: the GO enrichment analysis for intersection targets between compound and OSCC-related targets. Table S8: the enriched KEGG pathways for intersection targets between compound and AD-related targets. Table S9: the results of molecular docking. [file 5597586.f1.zip › 5597586.f1/Supplementary File 6. Topological analysis of the Compound-Target-Disease network.pdf]

Table S6. Topological analysis of the Compound-Target-Disease network.

Name	AverageShortestPathLength	BetweennessCentrality	ClosenessCentrality	Degree
OSCC	1.25438596	0.32329829	0.7972028	86
baicalein	1.85087719	0.02147713	0.54028436	52
Norwogonin	1.85087719	0.02173719	0.54028436	52
wogonin	1.86842105	0.01957835	0.53521127	51
rivularin	1.86842105	0.02017596	0.53521127	51
oroxylina	1.86842105	0.02564781	0.53521127	51
Moslosooflavone	1.86842105	0.01991209	0.53521127	51
viscidulinII	1.86842105	0.02014716	0.53521127	51
Salvigenin	1.86842105	0.02059457	0.53521127	51
SkullcapflavoneII	1.86842105	0.02014736	0.53521127	51
Panicolin	1.86842105	0.02014736	0.53521127	51
5,2'-Dihydroxy-6,7,8-trimethoxyflavone	1.86842105	0.01928031	0.53521127	51
acacetin	1.88596491	0.01933963	0.53023256	50
5,8,2'-Trihydroxy-7-methoxyflavone	1.88596491	0.01914466	0.53023256	50
5,7,2',6'-Tetrahydroxyflavone	1.90350877	0.02360646	0.52534562	49
chrysin	1.90350877	0.03234767	0.52534562	49
Eriodyctiol(flavanone)	2.14912281	0.01923498	0.46530612	35
Dihydrobaicalin_qt	2.18421053	0.01518258	0.45783133	33
Carthamidin	2.18421053	0.01518258	0.45783133	33
5,7,4'-trihydroxy-6-methoxyflavanone	2.20175439	0.0132709	0.45418327	32
dihydrooroxylinA	2.20175439	0.0132709	0.45418327	32
5,7,4'-trihydroxy-8-methoxyflavanone	2.21929825	0.01244019	0.45059289	31
SBG	1.77192982	0.02670857	0.56435644	27
ACHE	1.77192982	0.02795886	0.56435644	27
ABCC1	1.80701754	0.00890697	0.55339806	25
ABCG2	1.80701754	0.00890697	0.55339806	25
CYP1B1	1.80701754	0.00890697	0.55339806	25
ESR2	1.80701754	0.00890697	0.55339806	25
MMP13	1.80701754	0.00890697	0.55339806	25
SRC	1.80701754	0.00890697	0.55339806	25
Alpinetin	2.34210526	0.0117711	0.42696629	24
ABCB1	1.8245614	0.00752406	0.54807692	24
CA9	1.8245614	0.00752406	0.54807692	24
CYP19A1	1.8245614	0.00837944	0.54807692	24
KDR	1.8245614	0.00837944	0.54807692	24

MMP2	1.84210526	0.00643384	0.54285714	23
APP	1.85964912	0.0060252	0.53773585	22
KIT	1.85964912	0.00656914	0.53773585	22
TERT	1.85964912	0.0059829	0.53773585	22
ALOX12	1.87719298	0.00484296	0.53271028	21
MET	1.9122807	0.00555591	0.52293578	19
Dihydrooroxylin	2.44736842	0.00390401	0.40860215	18
EGFR	1.92982456	0.00491746	0.51818182	18
MMP9	1.92982456	0.00255566	0.51818182	18
PTGS2	1.92982456	0.00491746	0.51818182	18
ALOX5	1.94736842	0.0018105	0.51351351	17
AURKB	1.94736842	0.0018105	0.51351351	17
CDK1	1.94736842	0.0018105	0.51351351	17
DAPK1	1.94736842	0.0018105	0.51351351	17
FLT3	1.94736842	0.0018105	0.51351351	17
GSK3B	1.94736842	0.0018105	0.51351351	17
IGF1R	1.94736842	0.0018105	0.51351351	17
MPO	1.94736842	0.0018105	0.51351351	17
PIK3R1	1.94736842	0.0018105	0.51351351	17
PTK2	1.94736842	0.0018105	0.51351351	17
SYK	1.94736842	0.0018105	0.51351351	17
TOP2A	1.94736842	0.0018105	0.51351351	17
AHR	1.96491228	0.00161321	0.50892857	16
AKT1	1.96491228	0.00345441	0.50892857	16
F2	1.96491228	0.0016458	0.50892857	16
INSR	1.96491228	0.00164488	0.50892857	16
MMP3	1.96491228	0.00155782	0.50892857	16
IKBKB	1.98245614	0.00142387	0.50442478	15
NOS2	1.98245614	0.00137101	0.50442478	15
NTRK2	1.98245614	0.00142387	0.50442478	15
PLG	1.98245614	0.00145594	0.50442478	15
PLK1	1.98245614	0.00137734	0.50442478	15
TYR	1.98245614	0.00146337	0.50442478	15
PIK3CG	2	0.00122584	0.5	14
MCL1	2.01754386	0.0010923	0.49565217	13
ODC1	2.03508772	0.0013084	0.49137931	12
CDK6	2.05263158	8.75E-04	0.48717949	11
CYP1A1	2.05263158	8.75E-04	0.48717949	11
ALK	2.07017544	6.92E-04	0.48305085	10
AR	2.07017544	7.16E-04	0.48305085	10
CFTR	2.07017544	7.16E-04	0.48305085	10
CXCR1	2.07017544	6.92E-04	0.48305085	10
PARP1	2.07017544	7.16E-04	0.48305085	10

TTR	2.07017544	7.16E-04	0.48305085	10
EDNRA	2.0877193	0.00115929	0.4789916	9
PPARG	2.0877193	0.00115929	0.4789916	9
PTGS1	2.0877193	0.00115929	0.4789916	9
RXRA	2.0877193	0.00115929	0.4789916	9
SHBG	2.0877193	0.00115929	0.4789916	9
TOP1	2.0877193	5.76E-04	0.4789916	9
APEX1	2.10526316	4.52E-04	0.475	8
DNMT1	2.10526316	8.87E-04	0.475	8
FGFR1	2.10526316	8.34E-04	0.475	8
SERPINE1	2.10526316	8.34E-04	0.475	8
STAT1	2.10526316	8.87E-04	0.475	8
Baicalin	2.64035088	0.00259293	0.37873754	7
AXL	2.12280702	3.75E-04	0.47107438	7
BCL2	2.12280702	5.91E-04	0.47107438	7
LCK	2.12280702	4.28E-04	0.47107438	7
MAPK14	2.12280702	5.91E-04	0.47107438	7
MMP14	2.12280702	5.91E-04	0.47107438	7
HIF1A	2.14035088	6.10E-04	0.46721311	6
coptisine	2.6754386	0.00113724	0.37377049	5
epiberberine	2.6754386	0.00113724	0.37377049	5
CTSB	2.15789474	4.25E-04	0.46341463	5
PTPN1	2.15789474	4.25E-04	0.46341463	5
CDC42	2.19298246	0.0014639	0.456	3
CYP2D6	2.19298246	0.0014639	0.456	3
RAC1	2.19298246	0.0014639	0.456	3
ALDH2	2.21052632	4.59E-04	0.45238095	2
CDK2	2.21052632	5.05E-05	0.45238095	2
DRD2	2.21052632	5.00E-05	0.45238095	2
FYN	2.21052632	4.96E-05	0.45238095	2
HNF4A	2.21052632	1.08E-04	0.45238095	2
IL2	2.21052632	4.59E-04	0.45238095	2
MAPK3	2.21052632	4.96E-05	0.45238095	2
PRKDC	2.21052632	4.96E-05	0.45238095	2
TNF	2.21052632	4.59E-04	0.45238095	2
VEGFA	2.21052632	1.08E-04	0.45238095	2
