# Supplementary material for: Exploring the Mechanism of Scutellaria baicalensis Georgi Efficacy against Oral Squamous Cell Carcinoma Based on Network Pharmacology and Molecular Docking Analysis
Source: Evid Based Complement Alternat Med. 2021 Jul 13;2021:5597586. doi: 10.1155/2021/5597586 (PMC8292061; doi:10.1155/2021/5597586)
Supplement: Supplementary Materials — Table S1: detailed information of active compounds in SBG. Table S2: target gene-related active compounds of SBG. Table S3: list of OSCC-related genes in the GeneCards database, OMIM, and TTD. Table S4: the putative targets of SBG against OSCC. Table S5: topological analysis of the PPI network. Table S6: topological analysis of the compound-target-disease network. Table S7: the GO enrichment analysis for intersection targets between compound and OSCC-related targets. Table S8: the enriched KEGG pathways for intersection targets between compound and AD-related targets. Table S9: the results of molecular docking. [file 5597586.f1.zip › 5597586.f1/Supplementary File 7. The GO analysis for intersection targets between compound and OSCC related targets.pdf]

Table S7. The GO enrichment (MF,BP and CC) analysis for intersection targets between compound (OB ≥ 30% and DL ≥ 0.18) and OSCC related targets.

GO	Category	Description	LogP	Enrichment	#GeneInHitList	#GeneInGOAndHitList
GO:0004672	MF	protein kinase activity	-28.25927086	16.92434807	86	30
GO:0016773	MF	phosphotransferase activity, alcohol group as acceptor	-26.10053198	14.2977289	86	30
GO:0016301	MF	kinase activity	-24.93050345	13.03784891	86	30
GO:0019904	MF	protein domain specific binding	-14.50196703	9.573871409	86	21
GO:0042803	MF	protein homodimerization activity	-12.88991956	9.31433875	86	19
GO:0019900	MF	kinase binding	-11.91824121	8.202547653	86	19
GO:0019901	MF	protein kinase binding	-10.73101668	8.283978555	86	17
GO:0008134	MF	transcription factor binding	-11.0009884	8.619466783	86	17
GO:0004713	MF	protein tyrosine kinase activity	-20.3934961	37.46896436	86	16
GO:0004674	MF	protein serine/threonine kinase activity	-12.47224237	11.91804587	86	16
GO:0019902	MF	phosphatase binding	-13.49901577	21.81263486	86	13
GO:0016491	MF	oxidoreductase activity	-6.277192147	5.590027956	86	13
GO:0003682	MF	chromatin binding	-6.547865831	6.553925394	86	12
GO:0004714	MF	transmembrane receptor protein tyrosine kinase activity	-16.11247806	55.94731105	86	11
GO:0019199	MF	transmembrane receptor protein kinase activity	-14.79617935	43.14009526	86	11
GO:0019903	MF	protein phosphatase binding	-11.93507516	24.03105978	86	11
GO:0048037	MF	cofactor binding	-6.335634281	7.03463243	86	11
GO:0008289	MF	lipid binding	-3.81882739	4.130858222	86	10
GO:0020037	MF	heme binding	-9.356153625	21.22901921	86	9
GO:0046906	MF	tetrapyrrole binding	-9.085514447	19.79462602	86	9
GO:0051427	MF	hormone receptor binding	-7.119283913	14.71238996	86	8
GO:0042562	MF	hormone binding	-9.075187986	26.04093023	86	8
GO:0016705	MF	oxidoreductase activity, acting on paired donors, with incorporation or reduction of molecular oxygen	-7.396568496	15.97603082	86	8
GO:0050839	MF	cell adhesion molecule binding	-3.720807144	5.046691906	86	8
GO:0004175	MF	endopeptidase activity	-4.169348769	5.865074377	86	8
GO:0070011	MF	peptidase activity, acting on L-amino acid peptides	-3.204402569	4.220572161	86	8
GO:0008233	MF	peptidase activity	-3.123268894	4.100933895	86	8
GO:0019838	MF	growth factor binding	-6.649043045	16.63198099	86	7

GO:0035257	MF	nuclear hormone receptor binding	-6.501718959	15.82348191	86	7
GO:0044389	MF	ubiquitin-like protein ligase binding	-4.261506237	7.210700618	86	7
GO:0046982	MF	protein heterodimerization activity	-4.218676224	7.098384409	86	7
GO:0030545	MF	receptor regulator activity	-2.887515936	4.267006358	86	7
GO:0043548	MF	phosphatidylinositol 3-kinase binding	-9.404523722	65.10232558	86	6
GO:0042277	MF	peptide binding	-3.347146044	6.141728828	86	6
GO:0033218	MF	amide binding	-2.92764312	5.099398871	86	6
GO:0004497	MF	monooxygenase activity	-6.1255325	19.14774282	86	6
GO:0005506	MF	iron ion binding	-5.133965845	12.93423687	86	6
GO:0042169	MF	SH2 domain binding	-8.536993668	47.63584799	86	6
GO:0004879	MF	nuclear receptor activity	-7.892820361	37.55903399	86	6
GO:0098531	MF	transcription factor activity, direct ligand regulated sequence-specific DNA binding	-7.892820361	37.55903399	86	6
GO:0002020	MF	protease binding	-5.377159047	14.2559837	86	6
GO:0005178	MF	integrin binding	-5.252369253	13.5629845	86	6
GO:0031625	MF	ubiquitin protein ligase binding	-3.504430296	6.575992483	86	6
GO:0017171	MF	serine hydrolase activity	-4.529464765	10.11953247	86	6
GO:0005126	MF	cytokine receptor binding	-3.717773285	7.206899511	86	6
GO:0004715	MF	non-membrane spanning protein tyrosine kinase activity	-6.520082015	35.38169869	86	5
GO:0051219	MF	phosphoprotein binding	-5.181946702	19.14774282	86	5
GO:0001085	MF	RNA polymerase II transcription factor binding	-4.063084082	11.22453889	86	5
GO:0140097	MF	catalytic activity, acting on DNA	-3.252452617	7.500267924	86	5
GO:0042623	MF	ATPase activity, coupled	-2.893800654	6.235854941	86	5
GO:0016887	MF	ATPase activity	-2.214794774	4.328612073	86	5
GO:0051117	MF	ATPase binding	-5.107958946	18.49497886	86	5
GO:0045296	MF	cadherin binding	-2.435656517	4.887561981	86	5
GO:0031072	MF	heat shock protein binding	-4.336473373	12.81541842	86	5
GO:0004222	MF	metalloendopeptidase activity	-4.674715655	15.06998277	86	5
GO:0004252	MF	serine-type endopeptidase activity	-3.727391167	9.517883857	86	5
GO:0008236	MF	serine-type peptidase activity	-3.526338745	8.611418728	86	5
GO:0008237	MF	metallopeptidase activity	-3.526338745	8.611418728	86	5
GO:0070851	MF	growth factor receptor binding	-4.120525855	11.54296553	86	5
GO:0008201	MF	heparin binding	-3.751161419	9.630521536	86	5
GO:0005539	MF	glycosaminoglycan binding	-3.104855064	6.955376665	86	5
GO:1901681	MF	sulfur compound binding	-2.871902427	6.164992953	86	5
GO:0008022	MF	protein C-terminus binding	-3.526338745	8.611418728	86	5
GO:0051721	MF	protein phosphatase 2A binding	-5.553579171	40.68895349	86	4
GO:0042826	MF	histone deacetylase binding	-3.35387977	11.32214358	86	4

GO:0035258	MF	steroid hormone receptor binding	-3.93806516	16.07464829	86	4
GO:0016712	MF	oxidoreductase activity, acting on paired donors, with incorporation or reduction of molecular oxygen, reduced flavin or flavoprotein as one donor, and incorporation of one atom of oxygen	-5.393450236	37.2013289	86	4
GO:0008395	MF	steroid hydroxylase activity	-5.247355626	34.26438188	86	4
GO:0001784	MF	phosphotyrosine residue binding	-5.070619912	31.00110742	86	4
GO:0045309	MF	protein phosphorylated amino acid binding	-4.664134276	24.56691531	86	4
GO:0031406	MF	carboxylic acid binding	-2.29420695	5.812707641	86	4
GO:0043177	MF	organic acid binding	-2.214973907	5.517146236	86	4
GO:0042626	MF	ATPase activity, coupled to transmembrane movement of substances	-3.339650741	11.22453889	86	4
GO:0043492	MF	ATPase activity, coupled to movement of substances	-3.339650741	11.22453889	86	4
GO:0015405	MF	P-P-bond-hydrolysis-driven transmembrane transporter activity	-3.256980467	10.67251239	86	4
GO:0015399	MF	primary active transmembrane transporter activity	-3.243632715	10.585744	86	4
GO:0005158	MF	insulin receptor binding	-6.153053391	56.6107179	86	4
GO:0046875	MF	ephrin receptor binding	-5.860035278	48.22394488	86	4
GO:0051879	MF	Hsp90 protein binding	-5.070619912	31.00110742	86	4
GO:0004712	MF	protein serine/threonine/tyrosine kinase activity	-4.949443591	28.93436693	86	4
GO:0005518	MF	collagen binding	-4.185677533	18.60066445	86	4
GO:0051213	MF	dioxygenase activity	-3.742336995	14.30820342	86	4
GO:0005516	MF	calmodulin binding	-2.468413415	6.510232558	86	4
GO:0070491	MF	repressing transcription factor binding	-2.809572959	13.19641735	86	3
GO:0030331	MF	estrogen receptor binding	-3.530632572	23.25083056	86	3
GO:0043621	MF	protein self-association	-3.138722287	17.13219094	86	3
GO:0043559	MF	insulin binding	-6.254822072	162.755814	86	3
GO:0043560	MF	insulin receptor substrate binding	-5.48053639	97.65348837	86	3
GO:0017046	MF	peptide hormone binding	-3.280645989	19.14774282	86	3
GO:0070330	MF	aromatase activity	-4.212469	39.06139535	86	3
GO:0019825	MF	oxygen binding	-3.731099835	27.12596899	86	3
GO:0033293	MF	monocarboxylic acid binding	-2.71239716	12.20668605	86	3
GO:0008559	MF	xenobiotic transmembrane transporting ATPase activity	-6.254822072	162.755814	86	3
GO:0015562	MF	efflux transmembrane transporter	-5.002479953	69.75249169	86	3

		activity				
GO:0042910	MF	xenobiotic transmembrane transporter activity	-4.212469	39.06139535	86	3
GO:0042887	MF	amide transmembrane transporter activity	-3.255799962	18.77951699	86	3
GO:0015238	MF	drug transmembrane transporter activity	-2.289128086	8.641901626	86	3
GO:0070888	MF	E-box binding	-3.306013571	19.53069767	86	3
GO:0035173	MF	histone kinase activity	-4.73396897	57.44322845	86	3
GO:0004693	MF	cyclin-dependent protein serine/threonine kinase activity	-4.015284938	33.67361668	86	3
GO:0097472	MF	cyclin-dependent protein kinase activity	-4.015284938	33.67361668	86	3
GO:0030332	MF	cyclin binding	-3.970491313	32.55116279	86	3
GO:0001618	MF	virus receptor activity	-2.776270219	12.84914321	86	3
GO:0104005	MF	hijacked molecular function	-2.776270219	12.84914321	86	3
GO:0016702	MF	oxidoreductase activity, acting on single donors with incorporation of molecular oxygen, incorporation of two atoms of oxygen	-4.212469	39.06139535	86	3
GO:0016701	MF	oxidoreductase activity, acting on single donors with incorporation of molecular oxygen	-4.160187827	37.55903399	86	3
GO:0004601	MF	peroxidase activity	-3.255799962	18.77951699	86	3
GO:0016684	MF	oxidoreductase activity, acting on peroxide as acceptor	-3.16124275	17.43812292	86	3
GO:0016209	MF	antioxidant activity	-2.622756552	11.35505679	86	3
GO:0001223	MF	transcription coactivator binding	-4.015284938	33.67361668	86	3
GO:0001221	MF	transcription cofactor binding	-3.280645989	19.14774282	86	3
GO:0005496	MF	steroid binding	-2.401445935	9.480921201	86	3
GO:0005080	MF	protein kinase C binding	-3.184194154	17.7551797	86	3
GO:0097110	MF	scaffold protein binding	-3.073597777	16.2755814	86	3
GO:0051087	MF	chaperone binding	-2.389688817	9.389758497	86	3
GO:0007169	BP	positive regulation of transferase activity	-24.86318339	12.96859075	86	30
GO:0051347	BP	cellular response to nitrogen compound	-24.52732274	13.5629845	86	29
GO:1901699	BP	response to oxidative stress	-23.73678884	13.68517355	86	28
GO:0006979	BP	transmembrane receptor protein tyrosine kinase signaling pathway	-26.66377176	19.06467235	86	27
GO:0033674	BP	positive regulation of kinase activity	-23.33682468	14.2907544	86	27
GO:0010035	BP	response to inorganic substance	-22.89036377	14.90018015	86	26
