# Supplementary material for: Exploring the Mechanism of Scutellaria baicalensis Georgi Efficacy against Oral Squamous Cell Carcinoma Based on Network Pharmacology and Molecular Docking Analysis
Source: Evid Based Complement Alternat Med. 2021 Jul 13;2021:5597586. doi: 10.1155/2021/5597586 (PMC8292061; doi:10.1155/2021/5597586)
Supplement: Supplementary Materials — Table S1: detailed information of active compounds in SBG. Table S2: target gene-related active compounds of SBG. Table S3: list of OSCC-related genes in the GeneCards database, OMIM, and TTD. Table S4: the putative targets of SBG against OSCC. Table S5: topological analysis of the PPI network. Table S6: topological analysis of the compound-target-disease network. Table S7: the GO enrichment analysis for intersection targets between compound and OSCC-related targets. Table S8: the enriched KEGG pathways for intersection targets between compound and AD-related targets. Table S9: the results of molecular docking. [file 5597586.f1.zip › 5597586.f1/Supplementary File 8. The enriched KEGG pathways for intersection targets between compound and OSCC related targets.pdf]

Table S8. The enriched KEGG pathways for intersection targets between compound (OB ≥ 30% and DL ≥ 0.18) and OSCC related targets.

GO	Description	LogP	Enrichment	#Gene In Hit List	#Gene In GO And Hit List
hsa04151	PI3K-Akt signaling pathway	-25.865171	21.41523868	86	25
hsa04370	VEGF signaling pathway	-20.28317046	26.87710689	86	18
hsa04010	MAPK signaling pathway	-16.89052845	22.84292126	86	16
hsa04014	Ras signaling pathway	-16.86081444	49.44480424	86	12
hsa04015	Rap1 signaling pathway	-16.03302765	55.08658318	86	11
hsa04510	Focal adhesion	-16.00803014	20.10882643	86	16
hsa05418	Fluid shear stress and atherosclerosis	-15.90645449	17.29280523	86	17
hsa01521	EGFR tyrosine kinase inhibitor resistance	-15.80404544	22.71011357	86	15
hsa05205	Proteoglycans in cancer	-15.03554896	28.59223759	86	13
hsa04520	Adherens junction	-14.91834149	44.2052828	86	11
hsa05203	Viral carcinogenesis	-10.28675818	36.67736652	86	8
hsa04810	Regulation of actin cytoskeleton	-8.012530701	14.94696251	86	9
hsa04360	Axon guidance	-7.734630663	13.88438223	86	9
hsa05120	Epithelial cell signaling in Helicobacter pylori infection	-7.653201488	23.25083056	86	7
hsa04912	GnRH signaling pathway	-7.33167273	12.46640277	86	9
hsa05131	Shigellosis	-7.179364056	28.72161423	86	6
hsa04670	Leukocyte transendothelial migration	-7.018535401	18.8312512	86	7
hsa05100	Bacterial invasion of epithelial cells	-6.595844593	22.97729138	86	6
hsa04144	Endocytosis	-5.716162288	9.609199348	86	8
hsa05132	Salmonella infection	-4.945308889	17.13219094	86	5
hsa04530	Tight junction	-2.6415624	7.274002858	86	4
hsa01522	Endocrine resistance	-17.55817128	44.07969961	86	13
hsa04926	relaxin signaling pathway	-12.37340259	26.32814637	86	11
hsa05219	Bladder cancer	-10.15276536	51.7859408	86	7
hsa04915	Estrogen signaling pathway	-9.13804408	20.06578528	86	9
hsa04540	Gap junction	-5.060114858	18.08397933	86	5
hsa04921	Oxytocin signaling pathway	-3.84943328	10.10905677	86	5
hsa05215	Prostate cancer	-17.19953684	41.48677611	86	13
hsa05167	kaposi sarcoma-associated herpesvirus infection	-16.47588304	25.16842484	86	15
hsa04933	AGE-RAGE signaling pathway in diabetic complications	-15.20569303	36.50597696	86	12
hsa04380	Osteoclast differentiation	-13.81027229	28.10172327	86	12
hsa05163	human cytomegalovirus infection	-13.68210293	18.98817829	86	14

hsa04625	c-type lectin receptor signaling pathway	-13.5386567	33.46381221	86	11
hsa04660	T cell receptor signaling pathway	-13.22877084	31.40901673	86	11
hsa05212	Pancreatic cancer	-13.10813777	39.69653999	86	10
hsa04066	HIF-1 signaling pathway	-13.01968642	30.08931014	86	11
hsa05160	Hepatitis C	-12.78517943	23.11325169	86	12
hsa05165	human papillomavirus infection	-12.64944326	13.87123414	86	15
hsa05161	Hepatitis B	-12.54463367	22.06858494	86	12
hsa04062	Chemokine signaling pathway	-11.86182286	19.33732443	86	12
hsa05230	Central carbon metabolism in cancer	-11.61401663	37.55903399	86	9
hsa05226	gastric cancer	-11.53590199	22.1026414	86	11
hsa04664	Fc epsilon RI signaling pathway	-11.51181057	36.62005814	86	9
hsa04072	Phospholipase D signaling pathway	-11.50662875	21.96704237	86	11
hsa04932	Non-alcoholic fatty liver disease	-11.33493056	21.18714738	86	11
hsa04722	Neurotrophin signaling pathway	-11.27088361	26.25093773	86	10
hsa04611	Platelet activation	-11.09814542	25.23345953	86	10
hsa05142	Chagas disease	-10.35165885	27.37948272	86	9
hsa05224	Breast cancer	-10.11208258	20.09331036	86	10
hsa04917	Prolactin signaling pathway	-10.09083116	34.72124031	86	8
hsa05211	Renal cell carcinoma	-9.773558932	31.75723199	86	8
hsa05218	Melanoma	-9.730619413	31.37461474	86	8
hsa04071	Sphingolipid signaling pathway	-9.648646709	22.88753634	86	9
hsa04210	Apoptosis	-9.008128807	19.40135531	86	9
hsa04620	Toll-like receptor signaling pathway	-8.71179878	23.46029751	86	8
hsa05221	Acute myeloid leukemia	-8.685372254	32.55116279	86	7
hsa05223	Non-small cell lung cancer	-8.685372254	32.55116279	86	7
hsa05170	human immunodeficiency virus 1 infection	-8.644437643	14.21448157	86	10
hsa04919	thyroid hormone signaling pathway	-8.35690017	21.17148799	86	8
hsa05164	Influenza A	-8.293427357	16.09672885	86	9
hsa05225	hepatocellular carcinoma	-8.293427357	16.09672885	86	9
hsa04662	B cell receptor signaling pathway	-8.23468531	28.13063451	86	7
hsa04140	Autophagy - animal	-8.114227613	19.72797745	86	8
hsa04012	ErbB signaling pathway	-7.779744835	24.24022761	86	7
hsa05210	Colorectal cancer	-7.779744835	24.24022761	86	7
hsa05166	Human T-cell leukemia virus 1 infection	-7.596048182	11.03429247	86	10
hsa04150	mTOR signaling pathway	-7.501863462	16.48160141	86	8
hsa04931	insulin resistance	-7.223442832	20.16443713	86	7
hsa04725	Cholinergic synapse	-7.170793659	19.81375126	86	7
hsa05214	Glioma	-6.819549393	25.03935599	86	6
hsa04650	Natural killer cell mediated cytotoxicity	-6.420950644	15.39582024	86	7
hsa04550	Signaling pathways regulating pluripotency of stem cells	-6.32323182	14.89268886	86	7
hsa04910	Insulin signaling pathway	-6.285103546	14.70052513	86	7
hsa04666	Fc gamma R-mediated phagocytosis	-6.02705224	18.42518649	86	6

hsa04930	Type II diabetes mellitus	-6.008535986	28.06134723	86	5
hsa05231	Choline metabolism in cancer	-6.003052891	18.25298848	86	6
hsa05213	Endometrial cancer	-5.694147056	24.29191253	86	5
hsa01524	Platinum drug resistance	-5.508614473	22.29531698	86	5
hsa05220	Chronic myeloid leukemia	-5.258775274	19.84826999	86	5
hsa04024	cAMP signaling pathway	-5.195852793	10.08221856	86	7
hsa04371	Apelin signaling pathway	-4.048992876	11.14765849	86	5
hsa04261	Adrenergic signaling in cardiomyocytes	-4.035006375	11.07182408	86	5
hsa04022	cGMP-PKG signaling pathway	-3.703924925	9.407850518	86	5
hsa04960	Aldosterone-regulated sodium reabsorption	-3.626788113	25.03935599	86	3
hsa05222	Small cell lung cancer	-12.26412608	32.87996241	86	10
hsa05146	Amoebiasis	-3.472776392	12.16865899	86	4
hsa04668	TNF signaling pathway	-11.60147835	28.30535895	86	10
hsa05145	Toxoplasmosis	-11.56340111	28.06134723	86	10
hsa04657	IL-17 signaling pathway	-10.82323743	30.8379437	86	9
hsa04659	Th17 cell differentiation	-10.17165649	26.15718439	86	9
hsa05152	Tuberculosis	-8.090524156	15.25835756	86	9
hsa05140	Leishmaniasis	-6.595844593	22.97729138	86	6
hsa04658	Th1 and Th2 cell differentiation	-6.228230745	19.92928334	86	6
hsa04621	NOD-like receptor signaling pathway	-5.754012274	12.25043761	86	7
hsa05014	Amyotrophic lateral sclerosis	-4.538196169	22.84292126	86	4
hsa05133	Pertussis	-3.93806516	16.07464829	86	4
hsa05169	Epstein-Barr virus infection	-11.10426328	14.15267947	86	13
hsa05162	Measles	-10.21954085	20.60200177	86	10
hsa04630	JAK-STAT signaling pathway	-5.997954994	13.3250374	86	7
hsa05168	Herpes simplex virus 1 infection	-5.066207814	5.812707641	86	10
hsa05206	MicroRNAs in cancer	-10.96063048	13.78388001	86	13
hsa04068	foxo signaling pathway	-10.83614548	23.75997284	86	10
hsa04914	Progesterone-mediated oocyte maturation	-8.971995542	25.28245654	86	8
hsa04218	cellular senescence	-7.196027692	15.05256083	86	8
hsa04114	Oocyte meiosis	-6.804679497	17.52754919	86	7
hsa04110	Cell cycle	-5.470578904	14.79598309	86	6
hsa04115	p53 signaling pathway	-5.338662105	20.60200177	86	5
hsa04934	cushing syndrome	-4.82592001	11.42146063	86	6
hsa04913	Ovarian steroidogenesis	-9.498713692	42.19595177	86	7
hsa00380	Tryptophan metabolism	-3.306013571	19.53069767	86	3
hsa00140	Steroid hormone biosynthesis	-3.052656084	16.00876859	86	3
hsa00980	Metabolism of xenobiotics by cytochrome P450	-2.776270219	12.84914321	86	3
hsa05204	Chemical carcinogenesis	-2.580431803	10.97230206	86	3
hsa05202	Transcriptional misregulation in cancer	-7.861568673	14.36080711	86	9
hsa01523	Antifolate resistance	-7.411110846	52.50187547	86	5
hsa04064	NF-kappa B signaling pathway	-7.387579733	21.29515323	86	7

hsa04920	Adipocytokine signaling pathway	-4.137698975	18.08397933	86	4
hsa04622	RIG-I-like receptor signaling pathway	-2.8438539	13.5629845	86	3
hsa04928	parathyroid hormone synthesis, secretion and action	-7.223442832	20.16443713	86	7
hsa05216	Thyroid cancer	-3.626788113	25.03935599	86	3
hsa04726	Serotonergic synapse	-7.068372866	19.14774282	86	7
hsa00590	Arachidonic acid metabolism	-4.365892736	20.66740495	86	4
hsa04714	thermogenesis	-2.814879612	5.983669631	86	5
hsa05016	Huntington disease	-2.307888443	5.865074377	86	4
hsa04152	AMPK signaling pathway	-6.827644913	17.66342167	86	7
hsa04923	Regulation of lipolysis in adipocytes	-6.008535986	28.06134723	86	5
hsa04211	Longevity regulating pathway	-4.655366735	14.93172605	86	5
hsa04213	Longevity regulating pathway - multiple species	-4.393366363	21.00075019	86	4
hsa04060	Cytokine-cytokine receptor interaction	-6.109452688	8.931721497	86	9
hsa05323	Rheumatoid arthritis	-2.449751059	9.863988724	86	3
hsa04640	Hematopoietic cell lineage	-2.289128086	8.641901626	86	3
hsa02010	ABC transporters	-4.873337866	27.70311727	86	4
hsa04976	Bile secretion	-4.068330915	17.36062016	86	4
hsa05010	Alzheimer disease	-4.491927902	9.964641671	86	6
hsa04916	Melanogenesis	-3.504005311	12.40044297	86	4
hsa04750	inflammatory mediator regulation of trp channels	-3.519861459	12.519678	86	4
hsa00330	Arginine and proline metabolism	-2.991956685	15.25835756	86	3
hsa04728	Dopaminergic synapse	-3.128576089	9.863988724	86	4
hsa05321	Inflammatory bowel disease	-2.934215425	14.57514752	86	3
hsa04217	necroptosis	-2.741633474	7.750276855	86	4
hsa04610	Complement and coagulation cascades	-2.580431803	10.97230206	86	3
