# Supplementary material for: Exploring the Mechanism of Scutellaria baicalensis Georgi Efficacy against Oral Squamous Cell Carcinoma Based on Network Pharmacology and Molecular Docking Analysis
Source: Evid Based Complement Alternat Med. 2021 Jul 13;2021:5597586. doi: 10.1155/2021/5597586 (PMC8292061; doi:10.1155/2021/5597586)
Supplement: Supplementary Materials — Table S1: detailed information of active compounds in SBG. Table S2: target gene-related active compounds of SBG. Table S3: list of OSCC-related genes in the GeneCards database, OMIM, and TTD. Table S4: the putative targets of SBG against OSCC. Table S5: topological analysis of the PPI network. Table S6: topological analysis of the compound-target-disease network. Table S7: the GO enrichment analysis for intersection targets between compound and OSCC-related targets. Table S8: the enriched KEGG pathways for intersection targets between compound and AD-related targets. Table S9: the results of molecular docking. [file 5597586.f1.zip › 5597586.f1/Supplementary File 9. The results of molecular docking.pdf]

Table S9.The results of molecular docking.

	Molecular name	CAS ID	Core gene docking score（kcal/mol）				
			**MAPK3**	**VEGFA**	**SRC**	**AKT1**	**PIK3R1**
			PDB ID(4QTB)	PDB ID(3QTK)	PDB ID(1FMK)	PDB ID(4GV1)	PDB ID(6D85)
**Core**	Baicalein	491-67-8	-9.1	-8	-8.5	-8.1	-7.3
	Norwogonin	4443/9/8	-9.3	-8.1	-8.1	-8	-7
	Oroxylin-a	480-11-5	-9.1	-8.1	-7.9	-8.1	-6.9
	Salvigenin	19103-54-9	-9.4	-7.6	-8.1	-7.9	-6.7
	Rivularin	70028-59-0	-7.9	-7.7	-7.8	-7.6	-7.1
	Viscidulin II	92519-93-2	-8	-8.1	-7.4	-7.6	-6.8
	Wogonin	632-85-9	-7.9	-7.7	-7.8	-7.6	-6.8
	Panicolin	41060-16-6	-7.6	-7.8	-7.7	-7.5	-6.9
	Skullcapflavone II	55084-08-7	-8	-7.9	-7.3	-7.3	-6.9
	Moslosooflavone	3570-62-5	-6.9	-7.7	-7.7	-7.7	-6.8
**Positive drugs**	5-flourouracil	51-21-8	-6.3	-6.9	-6.9	-6.9	-7
	Cisplatin	15663-27-1	-12.3	-1.3	-1.4	-1.8	-1.4
